# From industry to consumer use: unraveling PFAS sources, bioaccumulation, and fluxes in a year-long catchment monitoring study

**DOI:** 10.1007/s10653-026-03200-0

**Published:** 2026-04-30

**Authors:** Yuheng Chen, Shanqi Zhou, Tianyi Li, Yulin Chen, Zulin Zhang

**Affiliations:** 1 Hubei Key Laboratory of Mineral Resources Processing and Environment, School of Resources and Environmental Engineering, Wuhan University of Technology, Wuhan, 430070 China; 2 Xianghu Laboratory, Hangzhou, 311231 China; 3 The James Hutton Institute, Aberdeen, AB15 8QH UK

**Keywords:** PFAS, Fluxes, Yangtze river, Bioaccumulation, Risk assessment

## Abstract

This study simultaneously determined the sources, bioaccumulation, and fluxes of 15 PFAS in surface water, sediment, and wild fish from the Wuhan section of the Yangtze River (WYR) at various locations during summer and winter. The results showed that the average concentrations of Σ_15_ PFAS in water, sediment and wild fish were 11.56 ng/L, 0.37 ng/g dw, and 11.91 ng/g dw, respectively. Source analysis using the Unmix model identified metal plating (41%) as the largest contributor among the three main sources. The annual load of PFAS in the WYR was estimated to be up to 7.15 t/a, and the WYR contributed 2.72 t/a to the Yangtze River. Health risk based on fish muscle suggested relatively low risk under Texas Commission on Environmental Quality (TCEQ) reference values, although the estimated weekly intake of the sum of PFOA, PFNA, PFHxS, and PFOS exceeded the tolerable intake value established by European Food and Safety Authority (EFSA). Our results provide data to address gaps in the understanding of PFAS sources, fluxes, and risk in the WYR, underscoring the need for further management of PFAS use and production in the area.

**Supplementary Information:**

The online version contains supplementary material available at 10.1007/s10653-026-03200-0.

## Introduction

Per- and polyfluoroalkyl substances (PFAS) are anthropogenic chemicals that have been widely used in textiles, lubricants, surfactants, food packaging, nonstick coatings, electronics, fire-extinguishing foams, and various other fields (Chen et al., 2023; Dai et al., 2025; Hu et al., 2016) due to their extreme thermal and chemical stability, and hydro- and lipophobic properties (Wang et al., 2013). However, during manufacture, use, and disposal, PFAS are easily released into the environment, and have been frequently detected in oceans (Boitsov, 2024), polar regions (Zhang et al., 2019), wildlife (Ahrens et al., 2015; Lohmann, 2024), and even in humans (Ehsan, 2023). They have been characterized as global environmental contaminants. Experimental data indicated that short-chain PFAS exhibit lower hydrophobicity, lower bioaccumulation potential, and lower acute toxicity than long-chain PFAS. However, PFAS as a class have been associated with a wide range of adverse health effects, including thyroid disruption, ulcerative colitis, high cholesterol, decreased immune responsiveness, kidney and testicular cancer (Fenton et al., 2021). This highlights the potential risk posed by the widespread presence of PFAS in environmental media. PFOA, PFOS, PFHxS and their salts have been listed in Annex A and B of the Stockholm Convention and have also been added to the list of priority new pollutants in China. PFAS are garnering increasing global attention (Lin et al., 2022). Therefore, monitoring PFAS is essential to provide a scientific basis for ecological management.

Hubei Province, located in central China, has been one of the main hubs of the fluorochemical industry since the 1960s, with six relevant producers accounting for 60% of China’s PFOSF (perfluorooctane sulfonyl fluoride) production. Wuhan, the industrial center of Hubei Province, has a well-developed industrial system, including steel, automotive, chemical, metallurgy, textiles, and manufacturing sectors, many of which have historically used PFAS-related products and processes (Tan, 2018; Wang et al., 2013). This intensive industrial activity has likely contributed to regional PFAS contamination. Notably, a major fluorochemical plant in Hubei closed in 2021 has left significant legacy contamination, with river water Σ_11_ PFAS concentrations reaching up to 23,118 ng/L and groundwater up to 67,937.9 ng/L near the site (Xu et al., 2025). Similarly, investigations at five former PFOS production sites in Hubei revealed extremely high on-site soil PFOS levels ranging from 392 ng/g to 7.78 × 10^5^ ng/g and groundwater concentrations up to 1.74 × 10^6^ ng/L (Jiang et al., 2025). The highest concentrations of PFOS (4.962 × 10^6^ ng/g dw) and PFOA (1.60×10^5^ ng/g dw) were detected in factory dust in Hubei Province (Wang et al., 2010). Considering the persistent PFAS, these industries may significantly contribute to the PFAS pollution burden in the regional environment.

The Yangtze River flows through Wuhan, where anthropogenic activities have the potential to contaminate its waters. Pan et al. (2014a) found that the load of PFAS in the Yangtze River was 20,900 kg/a, with Wuhan and Ezhou as major contributors. The Han River in Wuhan, which eventually drained into the Yangtze River, showed widespread PFAS pollution (Σ_8_ PFAS: 8.9–568 ng/L) (Wang et al., 2013). Hua et al. (2021) reported that Σ_13_ PFAS in sediment in the middle and lower reaches of the Yangtze River (Nanjing, Yangzhou, Taizhou, etc.) ranged from 13.83 to 20.33 ng/g dw. Although PFAS contamination in the Yangtze River Basin has attracted increasing attention, previous studies have mainly focused on the middle and lower reaches with limited number of target compounds, particularly on PFOA and PFOS. Therefore, this study aimed to systematically investigate PFAS contamination in the Wuhan section of the Yangtze River.

On the other hand, people living along the Yangtze River mostly consume freshwater fish, which has been suggested as an important route of human exposure to PFAS (Falandysz et al., 2006). PFAS can be transferred through the food chain, leading to bioaccumulation in organisms at higher trophic levels. Consequently, this study also investigated the PFAS in fish muscle to assess the health risks associated with the consumption of wild fish. In addition, the Unmix receptor model was used to identify potential PFAS sources in the aquatic environment and estimate their relative contributions. The findings of this research provide valuable data to support the development of PFAS control and management strategies.

The primary objectives of our study were (1) to investigate the occurrence and spatiotemporal variation of 15 PFAS in WYR, (2) to estimate the annual flux of PFAS in WYR and assess their potential sources, (3) to investigate the bioaccumulation of PFAS in fish and explore the factors that may contribute to their presence, and (4) to evaluate the ecological and health risks associated with PFAS. This study provides an integrated assessment of 15 PFAS in surface water, sediment and wild fish from WYR at various locations during summer and winter.

## Materials and methods

### Chemicals and reagents

As shown in Table S1 (Supplementary material), target compounds in this study included 15 PFAS of two groups: 11 perfluoro-carboxylic acids (PFCA) and 4 perfluro-sulfonic acids (PFSA). All standards and mass-labeled standards were purchased from Wellington Laboratories (Guelph, Canada). Methanol and acetonitrile of HPLC grade were obtained from Merck (Darmstadt Germany), and distilled water was purchased from Watsons (Guangzhou, China). Ammonium acetate (NH_4_OAc) was supplied by ANPEL (Shanghai, China). Besides, tetrabutylammonium hydrogen sulfate (TBAHS) and methyl tert-butyl ether (MTBE) were purchased from CNW Technologies GmbH (Dusseldorf, Germany).

### Study area and sample collection

**Fig. 1:**
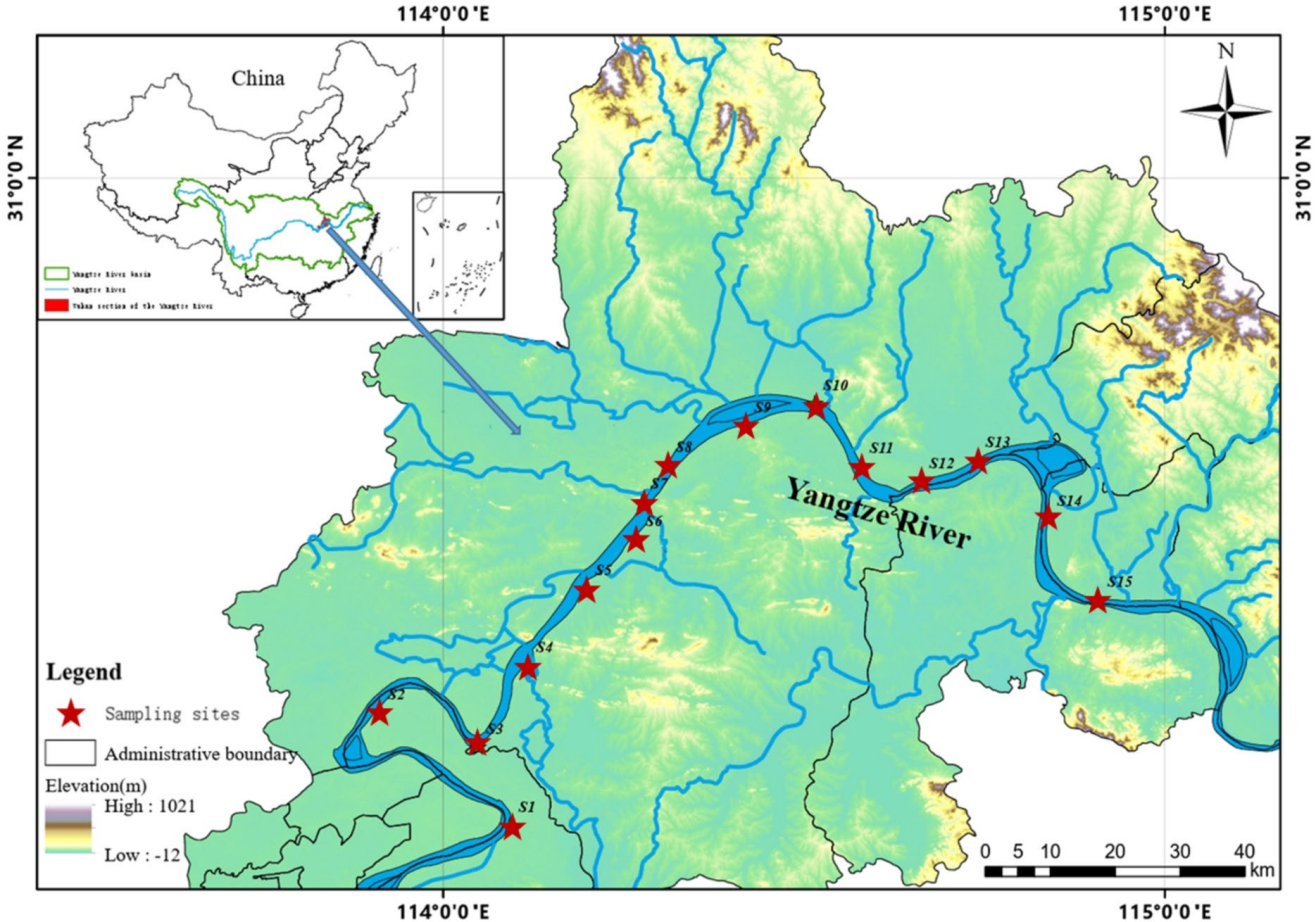
Map of the sampling locations in the Wuhan section of the Yangtze River

Samples were collected at 15 sites (S1-S15) from WYR in July 2022 (summer) and December 2022 (winter), as illustrated in Table S2 and S3 and Fig. 1. A total of 18 fish samples, 30 surface water samples (500 mL), and 30 sediment samples from the top 0–10 cm layer were collected and promptly transported to the laboratory. The detailed sampling method was shown in Text S1. In addition, two different monthly samples were collected in each season at S1, S8 and S15 (June/July 2022, Sep/Oct 2022, Dec 2022/Feb2023, March/April 2023) to estimate the annual PFAS fluxes. Seasonal categorization is based on the average temperature: summer (Jun-Aug), autumn (Sep-Nov), winter (Dec-Feb), and spring (Mar-May). Winter sampling, initially scheduled for Dec 2023 and Jan 2023, was postponed to Feb 2023 due to the COVID-19 Pandemic shutdown.

### Sample extraction and analysis

The sample extraction method in water, sediment, and fish was adapted from Zhao et al. (2014), Bao et al. (2009) and Taniyasu et al. (2005), respectively. 500 mL of filtered water samples spiked with 5 ng internal standard were enriched and eluted through PEP SPE column (500mg/6mL, Agela Technologies). Sediment samples weighing 5g were mixed with buffer and MTBE, followed by centrifugation and supernatant was extracted. Fish samples of 0.5 g were centrifuged, and the supernatant was collected and diluted with 300 mL of methanol. The subsequent analytical procedures were consistent with those applied to water samples. Detailed information was provided in Text S2.

The analysis of PFAS was conducted utilizing a PerkinElmer Qsight 210 triple quadrupole mass spectrometer (Waltham, USA), coupled with an LX50 high performance liquid chromatograph (HPLC) (Waltham, USA) in multiple reaction monitoring mode (MRM). The separation of target compounds was achieved using Agilent Zorbax-Eclipse Plus HPLC column (2.1*100 mm, 1.8 μm, San Jose, USA). A seven-point calibration curve (0.05, 0.1, 0.5, 1, 5, 10, 50 ng/mL) of each PFAS was prepared for quantification (R^2^ > 0.99) and recoveries of all target PFAS were in the range of 85.3–119.4% in water, 49.9–113.7% in sediment and 60.2–105.0% in fish. The limit of detection was defined as the analyte concentration corresponding to a signal-to-noise (S/N) ratio of 3:1, while the limit of quantification (LOQ) was defined as the analyte concentration corresponding to a S/N ratio of 10:1. The optimal conditions for MS/MS determination of the target compounds were shown in Table S1. The elution gradients, recoveries, and LOQ for the target compounds were summarized in Tables S4 and S5. Each batch of 9 samples contained one procedure blank and one solvent blank.

### Source analysis

Unmix is a receptor model used to identify potential contaminant sources in the environment through the analysis of extensive environmental monitoring data and requires only chemical species and their concentrations. It simplifies the massive monitoring data into combinations of species, referred to as source types and source contributions. The source type is characterized by a specific pattern of PFAS, allowing the model to match source contribution with observed concentrations. Previous studies had demonstrated the high accuracy of Unmix models, which not only offered reliable qualitative results but also compensated for the limitations in quantifying source contributions (Wallis, 2023). Therefore, EPA Unmix 6.0 was applied to assess the potential sources of PFAS in WYR in this study. Given the requirement for accurate information in source apportionment, five PFAS that were not detected were excluded from the analysis. As shown in Fig. 5, Unmix model predicted three main sources and identified the characteristic PFAS in each source. A weighted linear regression was used to fit the predicted and observed concentrations of total PFAS, as shown in Fig. S1.

### Mass flux estimation of PFAS

According to the established methodologies from previous studies, the fluxes of the target PFAS were estimated using two monitoring datasets per seasonal campaign collected from June 2022 to May 2023 (Niemi et al., 2022; Zhang et al., 2016). Table S6 and S7 summarize the flow rates and the detailed PFAS monitoring data obtained from S1, S8, and S15, with data below the LOD excluded from calculations. In addition to daily fluxes across various seasons, the annual fluxes, determined through the mean PFAS concentrations and average flow rates, were also analyzed at S1, S8, and S15, which respectively corresponded to the upper, middle, and lower reaches of WYR. The flux data obtained from these sites provided insights into the inflow loadings to WYR, as well as the mass of PFAS contributed by WYR to the Yangtze River.

## Risk assessment

### Ecological risk assessment in water

The risk quotient (RQ) method was employed as an assessment tool to evaluate the potential risks of PFAS in WYR. The RQ represents the ratio of the measured environmental concentration (MEC) obtained in this study to the predicted no-effect concentration (PNEC), as shown in the following equation (Eq. 1).

1 $${\mathrm{RQ}} = \frac{{{\mathrm{MEC}}}}{{{\mathrm{PNEC}}}}$$

2 $${\mathrm{PNEC}} = \frac{{{\mathrm{HC}}5}}{{{\mathrm{AF}}}}$$

Previous studies have reported the PNEC values for PFAS. Given the various sensitivity among organisms to PFAS, we denoted PNEC derived from species sensitivity distribution based on the species present in the WYR. The PNEC calculation was based on the hazardous concentration for 5% of species (HC5) along with an assessment factor (AF, here is 5) (Eq. 2) (Wang et al., 2024). Ecological data of PFAS was obtained from ECOTOX Database System. The RQ values for assessing the risks of PFAS in the water were divided into four levels: RQ < 0.01 (minimal risk), 0.01 ≤ RQ < 0.1 (low risk), 0.1 ≤ RQ < 1 (medium risk), and RQ ≥ 1 (high risk).

#### Health risk assessment in fish

In this study, the Hazard Ratio (HR) method was used to evaluate PFAS in wild fish, specifically to assess the potential health risk that may arise from the consumption of wild fish from WYR by local residents (Du et al., 2024). The daily intake and risk assessment of PFAS through fish consumption were calculated as follows:

3 $${\text{ADI = }}\frac{{{\mathrm{MEC}} \times {\mathrm{A}}{\mathrm{.C}}{.} \times (1 - \omega )}}{{{\mathrm{B}}{\mathrm{.W}}{.}}}$$

4 $${\mathrm{HR}} = \frac{{{\mathrm{ADI}}}}{{{\mathrm{RfD}}}}$$

where ADI (ng/kg/d) refers to the average daily intake of PFAS in the region. MEC is defined as the actual measured environmental concentration (ng/g dw) of PFAS in fish. B.W. and A.C. are the average body weight (kg) and the average daily fish intake (g/d ww) of Hubei province adults, respectively. ω is the average water content of fish (%), and RfD is the reference dose of PFAS (ng/kg/d).

In this study, A.C. is taken to be 46.63 g/d ww for adult (B.W., 63 kg body weight), which was according to the previous studies (Hu et al., 2020; Shi et al., 2009). ω takes on the average value of 75%, which is measured in the laboratory. RfD was obtained from *Texas Commission on Environmental Quality* publication in 2023, which provides the most comprehensive coverage of 15 PFAS studied in this work, as summarized in Table S9.

## Results and discussion

### Occurrence of PFAS in water and sediment

#### Occurrence of PFAS in water

Among the water samples, 10 target PFAS were detected, indicating that PFAS were widely distributed throughout WYR. The levels of total PFAS across the sampling sites in water ranged from 5.26 to 43.39 ng/L (mean: 11.56 ng/L) (Table 1 and S10). The concentrations of short-chain PFAS (C_4_-C_7_) ranged from 3.11 to 38.61 ng/L (mean: 8.52 ng/L), approximately three times higher than those of long-chain PFAS (C_8_-C_14_) (2.15–4.78 ng/L, mean: 3.04 ng/L). PFBS and PFBA were predominant in water, which contributed 32.4% and 16.4% of the total PFAS, respectively.

**Table 1:** The concentration (ng/L) and detection frequency of PFAS in water (n = 30), sediment (n = 30) and fish (n = 18) in Wuhan section of Yangtze River

	Compound	Water				Sediment				Fish			
		min	max	mean	DF	min	max	mean	DF	min	max	mean	DF
C_4_-C_7_ PFAS	PFBA	< LOD	12.56	1.90	60.0%	< LOD	0.029	0.0035	10.0%	< LOQ	0.098	0.035	38.9%
	PFPeA	< LOD	1.89	0.83	86.7%	< LOD	0.045	0.0063	3.3%	< LOQ	0.093	0.078	27.8%
	PFHxA	0.59	2.63	1.51	100.0%	< LOD	0.054	0.0076	6.7%	< LOQ	0.092	0.078	27.8%
	PFHpA	0.12	0.97	0.41	100.0%	< LOD	0.050	0.0065	3.3%	< LOQ	0.14	0.094	55.6%
	PFBS	0.92	35.50	3.74	100.0%	< LOD	0.061	0.010	96.7%	< LOQ	< LOQ	< LOQ	–
	PFHxS	< LOD	0.83	0.14	90.0%	< LOD	0.086	0.0066	33.3%	< LOQ	< LOQ	< LOQ	–
C_8-_C_14_ PFAS	PFOA	1.51	3.79	2.20	100.0%	< LOD	0.66	0.044	60.0%	< LOQ	5.01	1.91	94.4%
	PFNA	0.10	0.32	0.22	100.0%	< LOD	0.037	0.0098	20.0%	< LOQ	0.42	0.12	72.2%
	PFDA	< LOD	< LOD	< LOD	–	< LOD	0.048	0.0083	10.0%	0.086	1.79	0.62	100.0%
	PFUnDA	< LOD	< LOD	< LOD	–	< LOD	0.053	0.0093	10.0%	0.088	2.91	1.40	100.0%
	PFDoDA	< LOD	< LOD	< LOD	–	< LOD	< LOD	< LOD	–	< LOQ	0.64	0.26	83.3%
	PFTrDA	< LOD	< LOD	< LOD	–	< LOD	< LOD	< LOD	–	< LOQ	6.87	1.58	88.9%
	PFTeDA	< LOD	< LOD	< LOD	–	< LOD	< LOD	< LOD	–	< LOQ	0.90	0.35	72.2%
	PFOS	0.16	1.51	0.46	100.0%	< LOD	3.18	0.23	96.7%	0.98	11.23	5.02	100.0%
	PFDS	< LOD	0.13	0.028	3.3%	< LOD	0.0073	0.0051	3.3%	< LOQ	2.04	0.32	77.8%
Total PFAS	C_4_-C_7_ PFAS	3.11	38.61	8.52	89.4%	0.018	0.25	0.041	25.6%	0.27	0.38	0.32	25.0%
	C_8_-C_14_ PFAS	2.15	4.78	3.04	33.7%	0.037	3.88	0.32	22.2%	3.87	22.63	11.59	87.7%
	15PFAS	5.26	43.39	11.56	61.6%	< LOD	4.02	0.37	23.9%	4.20	23.00	11.91	62.6%

In recent years, short-chain PFAS have been frequently detected in surface water, likely due to their extensive use in manufacturing processes as alternatives to long-chain PFAS, which are also resistant to both natural (e.g., biodegradation, hydrolysis, and photolysis) and engineered (e.g., adsorption and oxidation) treatment processes (Bai & Son, 2021). In contrast, the long-chain PFAS contributed only 26.3% of the total PFAS, with PFOA representing 72.6% of this fraction. PFOA was banned a decade later than PFOS, which may explain why PFOA was detected with higher concentration than PFOS. PFOA and PFOS were used for over 30 years and exhibited high persistence. Even after the cessation of their production, these compounds continued to be frequently detected, albeit at lower concentrations (Barbosa et al., 2023), largely due to their inherent environmental persistence and partly because of the exemption of PFOS and PFOA in firefighting foam agents (Dauchy et al., 2017).

#### Occurrence of PFAS in sediment

The PFAS composition in sediment differed from that in water (Table 1 and S11). A total of 12 target compounds were detected except for PFDoDA, PFTrDA, and PFTeDA. The total PFAS concentrations in sediment ranged from < LOD to 4.02 ng/g dw in WYR, with an average concentration of 0.37 ng/g dw. PFOS (mean: 0.23 ng/g dw) and PFOA (mean: 0.044 ng/g dw) were predominant in sediment, contributing to 64.0% and 12.0% of the total PFAS, respectively. The levels of PFBS were lower than PFOS, ranging from < LOD to 0.061 ng/g dw (mean: 0.010 ng/g dw). Comparing with other studies, the total PFAS concentrations in sediment from WYR were lower than those observed in the Danube River in Europe, where the concentrations of 15 PFAS were up to 6.3 ng/g dw, with PFOS reaching 5.7 ng/g (Beškoski et al., 2013). However, they were similar to those detected in Dongshan Bay (Σ_25_ PFAS: 0.15–0.37 ng/g dw) (Lin et al., 2022).

Comparing the distribution of PFAS in water and sediment, the short-chain PFAS were predominant in water, whereas the long-chain PFAS were more prevalent in sediment than in water. This is likely because of the higher hydrophobicity and stronger sorption affinity of the long-chain PFAS to organic matters in sediment (Gan et al., 2022).

#### Partitioning behavior of PFAS between water and sediment

**Fig. 2:**
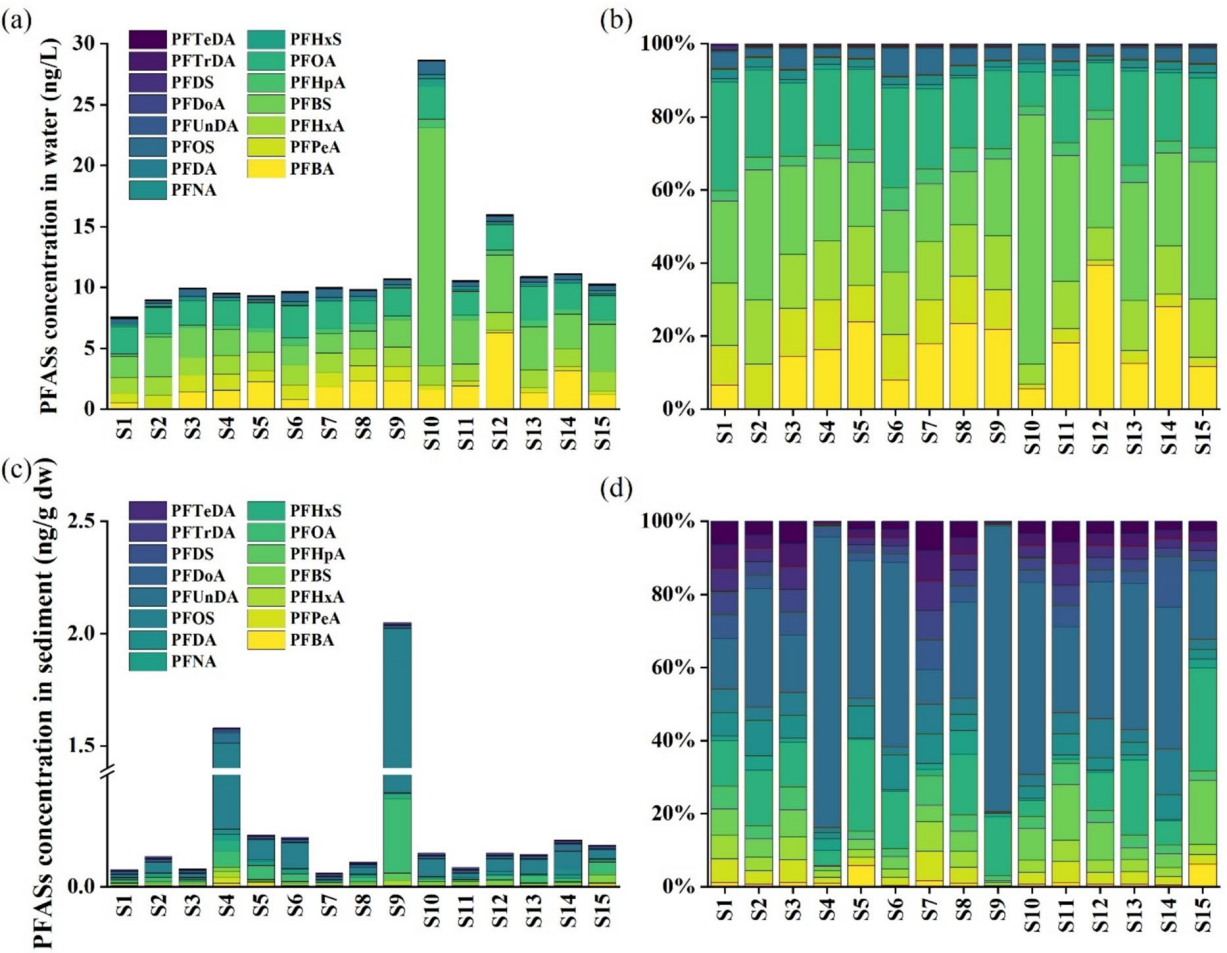
Concentration distribution and composition of PFAS in water **a**, **b**, sediment **c**, **d** from WYR

PFAS partitioning between water and sediment is essential for understanding the transport of PFAS in the environment. As shown in Fig. 2a and c, the patterns of PFAS concentration varied bettewn sediment and water. Figure 2c and d further revealed that long-chain PFAS tended to accumulate in sediment, whereas short-chain PFAS were more in water. To gain a clearer understanding of its partitioning, the sediment–water log K_oc_ values of individual PFAS were calculated using the equation in Text S3, as shown in Table S13.

**Fig. 3:**
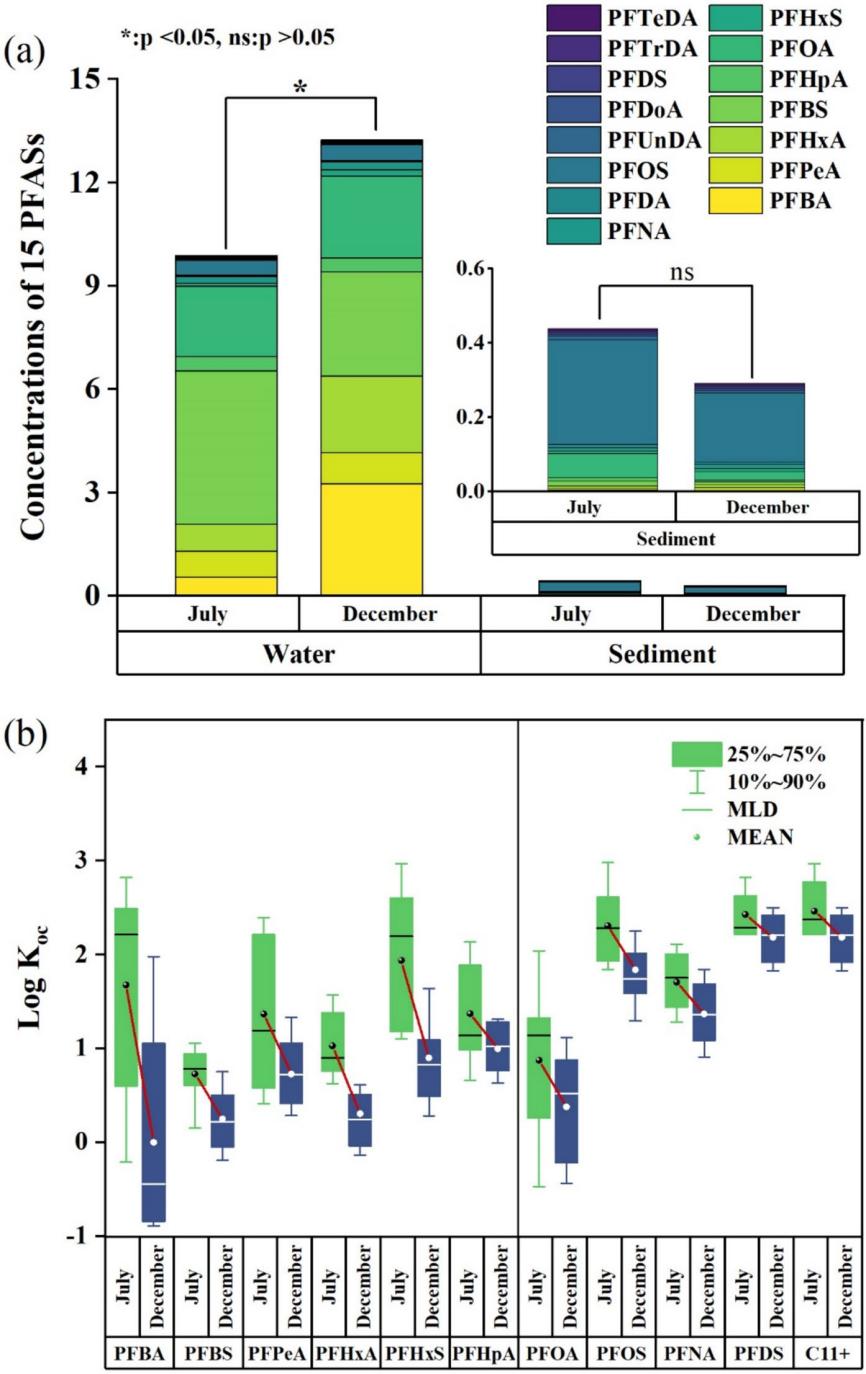
Seasonal variations of PFAS concentration in water (ng/L) and sediment (ng/g dw) (**a**), and the seasonal variations in sediment and water log Koc in WYR (**b**)

It was observed that with increasing carbon chain length, the log K_OC_ values showed a gradual upward trend (Fig. 3b). The results indicated that long-chain PFAS were more inclined to migrate to sediment while short-chain PFAS tended to migrate to water. Ahrens et al. (2016) also observed this in their study of Lake Tana in Ethiopia, speculating that it may be related to the greater hydrophobicity of long-chain PFAS compared to the relatively hydrophilic short-chain PFAS. Furthermore, different functional groups may also influence the partitioning of PFAS between water and sediment. In this study, the log K_OC_ of PFOS was greater than that of PFOA, and a similar trend was found in PFHxS and PFHxA, despite both having the same carbon chain length. This finding was consistent with Vierke’s study, which noted that solids adsorbed sulfonic acids more strongly than carboxylic acids (Vierke et al., 2014).

The properties of sediments also affected log K_OC_ in addition to the structure of PFAS. Higgins and Luthy (2006) found that the adsorption of PFAS on sediment was related to the properties of sediment and the electrochemical characteristics of water. In this study, the log K_OC_ of four substances (PFBS, PFBA, PFHxS, PFNA) with high DF (detection frequency) in both sediment and water had a significant correlation with the TOC of sediment (p < 0.05) (Fig. S3). However, some studies found no correlation between the log K_OC_ and TOC (Becker et al., 2010; Li et al., 2010). This indicated that the partitioning of PFAS in sediment and water may be influenced by a combination of factors within the aquatic environment (e.g., pH, ionic strength, microbial activity, hydraulic disturbances, salinity). Given this, the driving forces affecting the migratory capacity of PFAS require further in-depth study.

### Spatial–temporal distribution of PFAS in water and sediment

#### Spatial variations of PFAS

The spatial variations of PFAS concentrations were shown in Fig. 2a and c. Generally, in surface water, the concentrations of total PFAS exhibited minimal variation (Fig. 2a), except for S10 and S12, where PFAS-related industrial plants were clustered. For illustration, S10 (Σ_15_ PFAS: 28.69 ng/L) is located next to a large industrial park, where PFAS are widely used as hydrophobic and oleophobic agents (Lindstrom et al., 2011). S12 (Σ_15_ PFAS: 15.97 ng/L) is surrounded by lots of materials manufacturing bases, including those producing asphalt, shipbuilding materials, and aerospace components, all of which are significant applications for PFAS. The discharge of these industrial wastes likely contributed to the high concentrations of PFAS in waters at S10 and S12.

In sediment, higher concentrations were mainly observed at S4 (Σ_15_ PFAS: 1.58 ng/g dw) and S9 (Σ_15_ PFAS: 2.05 ng/g dw), while relatively low levels occurred at S1, S3, S7 and S11 (Σ_15_ PFAS < 0.10 ng/L), as shown in Fig. 3c. Unlike the relatively uniform pattern in water, sediment PFAS displayed stronger site-specific differences, suggesting that the concentrations of total PFAS were related to the properties of sediment in distinct sites. In this study, Spearman correlation analysis revealed significant positive correlations (*p* < 0.05) between the concentrations of total PFAS and TOC in sediment. This may be due to the fact that the low TOC content greatly weakens the adsorption of long-chain PFAS to the sediment, eventually leading to the low PFAS concentrations (Li et al., 2024b). Likewise, Chen et al. (2018) investigated the top soil of Fuxin Industrial Park and found that the TOC content was the primary factor controlling the adsorption rate of PFAS. In addition to the properties of the sediment samples, tributary inflows may be an important factor influencing PFAS concentrations. S7 is located downstream of a major tributary, the Han River, which flowed into the mainstream to dilute the concentrations of target compounds in water and accelerate the flow rate, leading to the weakening of the adsorption capacity of the sediment (Barbosa et al., 2023).

#### Seasonal variations of PFAS

In surface water, the concentrations of total PFAS showed significant seasonal variations (Wilcoxon rank sum test, *p* < 0.05), with winter concentrations (mean: 13.23 ng/L) being higher than those in summer (mean: 9.88 ng/L) (Fig. 3a and Table S15). Podder et al. (2021) found that the total concentrations of PFAS in surface water was lower in wet season due to the dilution. In our study, the average flow rate during summer was 30,845 m^3^/s, which was higher than in winter (9149 m^3^/s). Therefore, dilution due to runoff likely played a key role in the seasonal variation of PFAS in WYR. Lower water levels during the dry season tend to retain pollutants, resulting in relatively high concentrations.

Seasonal differences were also observed for the occurrence of PFBA, PFOA, PFHxA, PFNA, PFHxS (*p* < 0.05), for which the average concentrations in winter were higher than those in summer. Similarly, water flow rates at different times may influence such variations. In addition, it has been shown that the concentrations of PFAS in weathered textiles increased 5 to more than 100 fold (especially outdoor clothes), with PFHxA and PFOA being the most commonly detected PFAS in this process (Van Der Veen et al., 2020). Thus, the significant seasonal distribution of PFHxA and PFOA may be also attributed to the fact that humans are more inclined to wear rain- and wind-proof jackets in winter, which facilitates the release of PFAS into the environment (Schellenberger et al., 2022). Seasonal variations (winter > summer) in the concentrations of PFHxA and PFOA were also observed in European Elbe (Zhao et al., 2015). However, Li et al. (2024a) found no significant seasonal variations in water from the middle and lower reaches of the Yangtze River in 2014. Consequently, there was also regional variability in the seasonal variation of PFAS.

In sediment, no significant seasonal variations were observed (Wilcoxon rank sum test, *p* > 0.05), with PFOS and PFOA always being the dominant species (Table S16). This finding aligned with the study conducted by Chen et al. (2016) on Bohai Sea sediments, which reported no significant seasonal variations. Similarly, a study conducted on the Chaobai River in Beijing found that the total concentrations of 43 PFAS in the sediment were not significantly changed between August and November (Cai et al., 2022).

### Potential source of PFAS in the Wuhan section of the Yangtze river

**Fig. 4:**
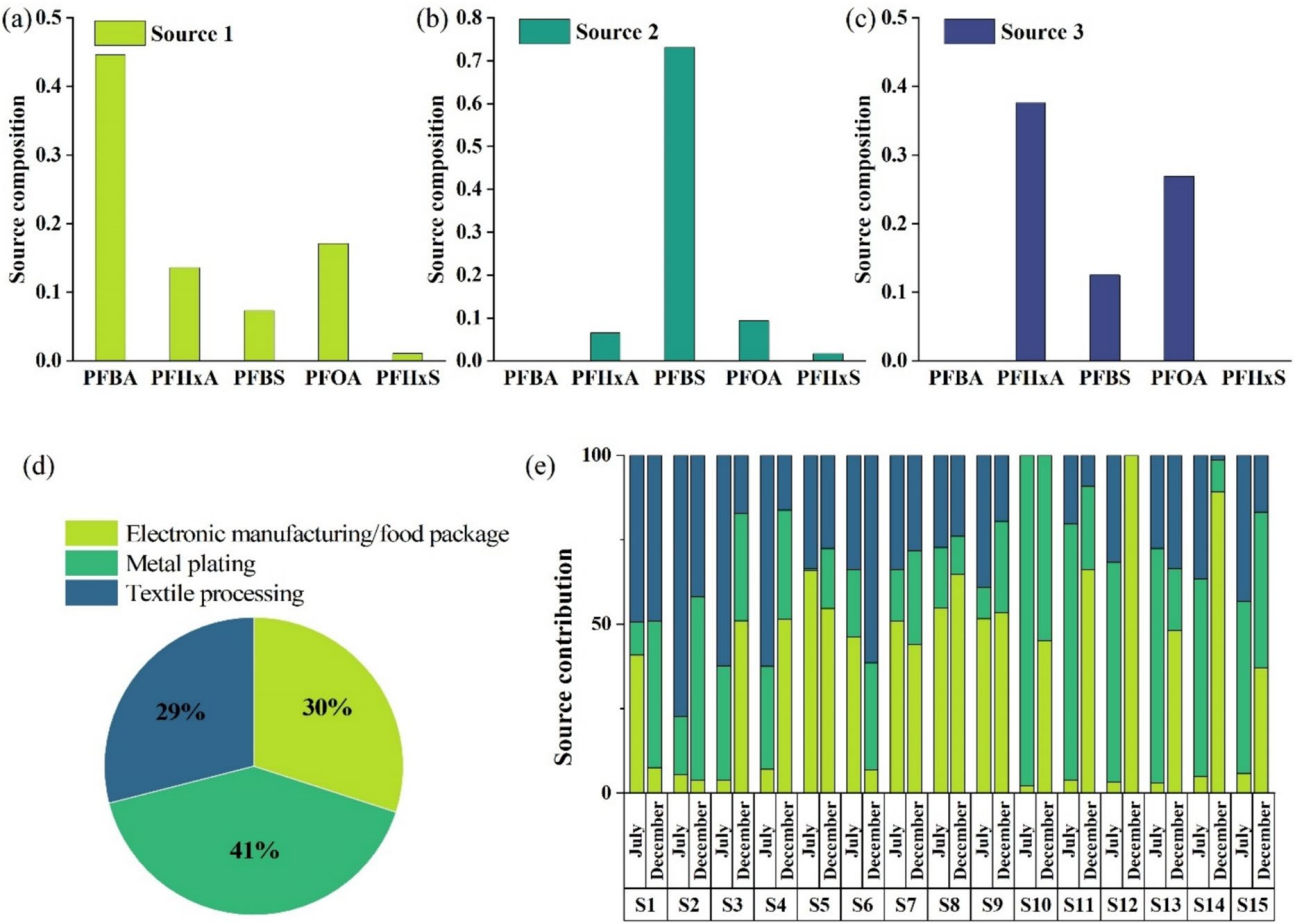
PFAS profiles apportioned to source 1 (**a**), source 2 (**b**), and source 3 (**c**), source composition (**d**), and source contribution (**e**) analyzed by Unmix model

Unmix model was employed to identify three main sources of PFAS in the water of WYR (Fig. 4). The predicted values of the model were in good agreement with the actual measured values of the samples (R^2^ = 0.98, *p* < 0.01), as shown in Fig. S1.

In source 1, PFBA was the most prominent compound, a short-chain PFAS commonly used as an alternative to PFOA. PFBA has been widely employed as a cleaning agent and etchant within electronic manufacturing processes. Its usage is linked to the optoelectronics sector, encompassing applications such as semiconductor chips and battery production (Hua et al., 2021; Tansel, 2022; Zarębska et al., 2024). According to local industrial statistics, multiple electronics processing enterprises are distributed in Wuhan (Wuhan Municipal Bureau of Statistics And Survey Office of the National Bureau of Statistics in Wuhan, 2023). In addition, PFBA was also widely detected in food package (Zafeiraki et al., 2014). Thus, source 1 was identified as electronic manufacturing/food package.

PFBS was relatively abundant in source 2, which was regarded as a lower toxicity alternative to PFOS due to its similar properties, but lower half-life, bioaccumulation potential and toxicity (Wallis, 2023). PFBS was primarily used as an atomizer in the metal plating industry (Xiao et al., 2012; Zhang et al., 2025), suggesting that source 2 was related to metal plating.

In source 3, the contribution of PFHxA and PFOA were relatively high. These substances were commonly used in textile, leather processing and other industries (Joerss et al., 2022; Zarębska et al., 2024), indicating that source 3 was linked to textile processing.

Figure 4d illustrated the relative contributions of the three sources to PFAS contamination, with source 2 (metal plating) being the main source and accounting for 41%, followed by source 1 (electronic manufacturing/food package) at 31%, and source 3 (textile processing) at 29%. This was consistent with the industrial activities in Wuhan, which is an important industrial and high-tech base in central China (Bao et al., 2009). Consistent with the present study, Hua et al. (2021) used the PMF model and identified that electroplating and fast-food packaging were the major sources in surface sediment from the middle and lower reaches of the Yangtze River. Another study also showed that the majority of PFAS was from the direct emissions from manufacturing processes in Tai Lake (Guo et al., 2015).

Spatially, the composition of PFAS pollution sources at each sampling point was shown in Fig. 4e. Source 1 (electronic manufacturing/food package) contributed to all sampling points, with its highest distribution in the middle and lower reaches of WYR, where industrial activities and human populations were concentrated. Previous studies had demonstrated that fast food package release volatile PFAS into the air (Zafeiraki et al., 2014), and the widespread usage and disposal of food packaging bags had led to the transfer of substantial amounts of PFAS into the environment. The increasing prevalence of take-out business in China may have exacerbated this contamination process. Source 2 (metal plating) was predominantly found in S10-S15 of WYR, particularly around the Qingshan District of Wuhan, where shipbuilding factory and steel mills were highly concentrated (Wuhan Municipal People’s Government, 2024). Source 3 (textile) was mainly located in the upper reaches of WYR, bordered by Xianning and Xiantao City within Hubei Province. According to the industrial distribution of Hubei Province, this region is chiefly characterized by the prominence of the apparel and textile sectors. Consequently, the pollution was likely related to the direct discharge of wastewater from textile mills which was widely distributed around the region.

In addition to the sources identified, long-range atmospheric transport and the indiscriminate disposal of carpet and clothing products may also contribute to PFAS emissions. An et al. (2021) evaluated the impact of non-point sources in Tai Lake and revealed that the effect of runoff was obvious while the effect of atmospheric deposition was weak. Additional effort is necessary, as identifying pollutant sources is a critical strategy for controlling emissions.

### Mass flux estimation of PFAS

The annual PFAS fluxes across monitoring sites were shown in Table S18. Estimated annual fluxes of total PFAS exhibited an upstream–downstream increasing gradient: 5.22 t/a (S1), 7.15 t/a (S8), and 7.95 t/a (S15) in the aqueous phase, indicative of the input sources along the WYR. Furthermore, the estimated WYR contribution to dissolved PFAS in the Yangtze River was 2.72 t/a (excluding migration and diffusion processes). Compared with the total annual flux of 18 PFAS to the sea (20.7 t/a, in both water and sediment phases) reported by Pan et al. (2014a) at Shanghai, the WYR contribution (2.72 t/a) may represent an appreciable proportion of PFAS transport to the whole Yangtze River. Li et al. (2024a) also estimated the annual mass load of PFAS in the lower of the Yangtze River to be 10.8 t/a (in both water and sediment phases), which was higher than the annual flux (S15: 7.95 t/a) in this study, indicating that the Yangtze River also receive external PFAS contamination from Wuhan to the downstream. As both Pan et al. (2014a) and Li et al. (2024a) were conducted within the Yangtze River, these studies provide regionally relevant comparisons for the present study. Compared with a recent study from another river system, the annual PFAS flux observed in this study was higher than the annual PFAS export reported for the River Mersey (68.1 kg/a) by Byrne et al. (2024). However, direct comparisons among studies should be interpreted with caution because the reported values may be influenced by differences in target PFAS, environmental matrices, hydrological conditions, watershed scale, and calculation methods. Overall, these results indicated substantial PFAS transport in the WYR, while also highlighting clear spatial variability along the river.

For individual compounds, the annual fluxes of PFBS exhibited progressive downstream increases from S1 to S15. However, PFHxA and PFOA demonstrated a decline at S8, followed by an increase at S15, while other compounds (PFBA, PFPeA, PFOS, etc.) rose at S8 and then dropped at S15. This spatial heterogeneity suggested differential industrial source distributions: industries related to PFHxA and PFOA were mainly concentrated in S9-S15 versus PFBA, PFPeA, PFOS-associated sources prevalent around S1-S8. Additionally, the processes of pollutant retention by sediment, wastewater plant discharge, and the atmospheric deposition also influenced the fluxes (Lin, 2022; Qiao et al., 2024). Among the 10 PFAS (> LOD), the annual fluxes of PFBS, PFHxA, and PFOA accounted for the largest share at S1, S8, and S15. Notably, PFBS contributed over 30% of the total annual flux at all 3 sites, ranging from 1.69 to 3.23 t/a.

Moreover, the average daily fluxes of 10 PFAS across four seasons were calculated in this study (Table S17). The average daily fluxes at all 3 sites were higher in summer (14.93–19.71 kg/d) than those in winter (9.06–11.67 kg/d), interestingly, showing an inverse pattern to the PFAS concentration trends (winter > summer) observed in Sect. “Occurrence of PFAS in water and sediment”. This may be caused by the seasonal fluctuations in the flow of WYR, which diluted PFAS during high-flow periods, emphasizing that concentration metrics alone inadequately reflect contaminant mass transfer. Comprehensive flux analysis therefore proves critical for accurate pollution assessment in WYR.

### Bioaccumulation of PFAS in wild fish

#### Occurrence of PFAS in fish

The concentrations of total PFAS in 18 wild fish muscle samples ranged from 4.20 to 23.00 ng/g dw (mean: 11.91 ng/g dw), PFOS contributed 42.2% to the total concentrations (Table S12). Except for PFBS and PFHxS, the remaining 13 PFAS were detected in fish samples, with the DF of PFDA, PFUnDA and PFOS reaching 100%. The DF for the other six long-chain PFAS were greater than 70.0%, while short-chain PFAS were below 40.0%, except for PFHpA (55.6%). Like the findings in sediment, long-chain PFAS were dominant, accounting for 97.4%. Lee et al. (2020) studied PFAS in the multimedium samples from Asan Lake, South Korea, and found that long-chain PFAS, especially PFOS, were dominant in the muscle of fish samples. Compared with previous studies, the concentrations of PFAS in wild fish from WYR in this study were higher than those of Westlake and Yen So Lake in Vietnam(Hoa et al., 2022) (Σ_17_ PFAS: 0.51–2.60 ng/g ww), and Lake Tana in Ethiopia (Ahrens et al., 2016) (Σ_26_ PFAS: < LOD-5.80 ng/g ww), but lower than those detected in the Jiulong River in China (Wang et al., 2022) (Σ_21_ PFAS: 25–100 ng/g ww), estuaries and coastal areas in South Korea (Naile et al., 2010) (PFOS: 0.26–612 ng/g), Kamo River. The concentrations were comparable to those observed in the West River and North River in China (Chen et al., 2021a, 2021b) (Σ_11_ PFAS: 1.87–28.40 ng/g dw), and in six major rivers in Korea (Lam et al., 2014) (mean: 6.13 ng/g ww).

**Fig. 5:**
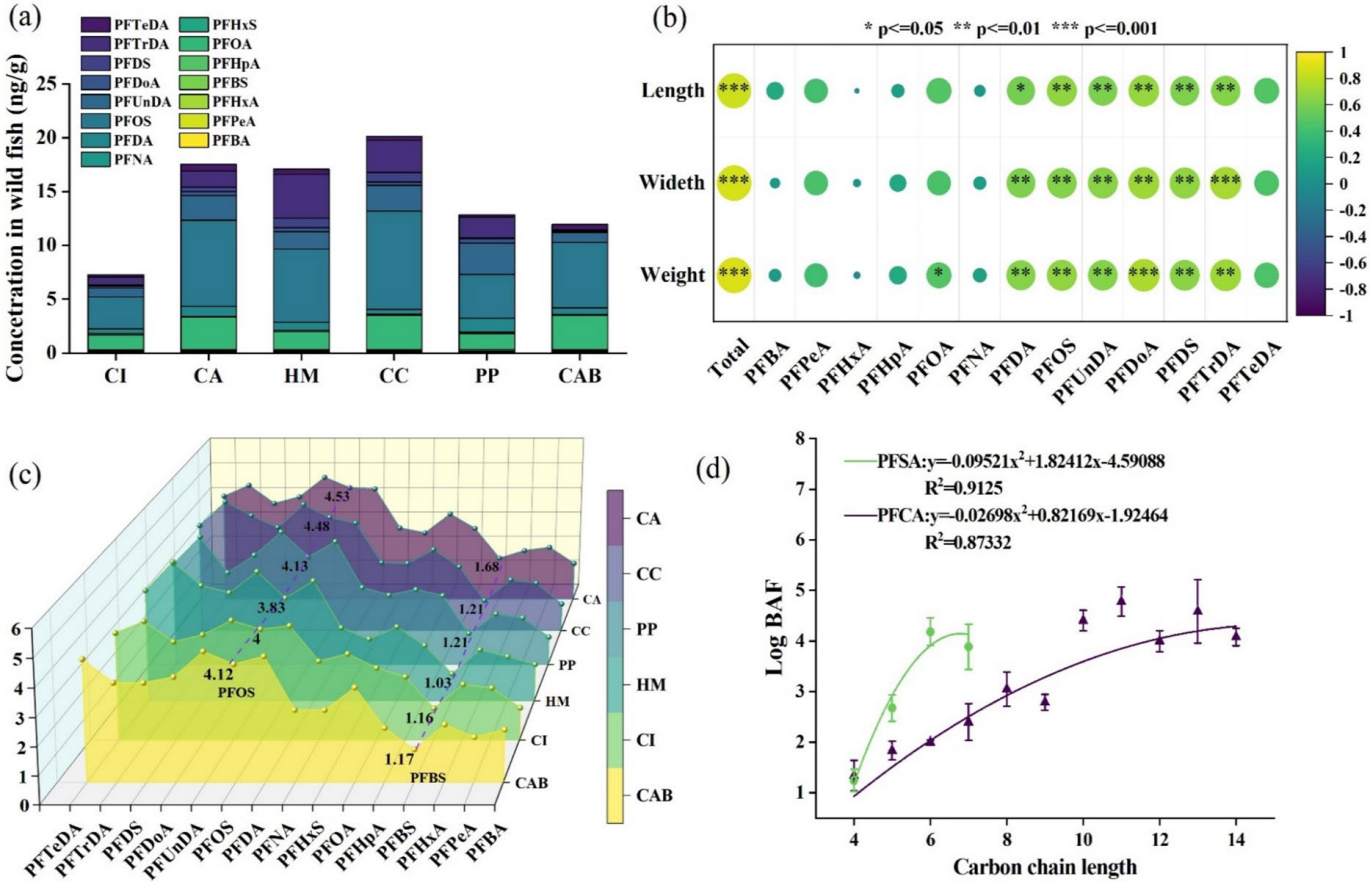
Concentration of PFAS in wild fish (**a**), the correlation between concentration and size of fish (**b**), the log BAF of wild fish from WYR (**c**), and the fit curves of log BAF and carbon chain length in PFCA and PFSA (**d**). (CA: *Carassius auratus*, CC: *Cyprinus carpio*, PP: *Parabramis pekinensis*, HM: *Hypophthalmichthys molitrix*, CI: *Ctenopharyngodon idellus*, CAB: *Culter alburnus basilewsky*)

Variations in dietary composition and quantity can lead to the development of distinct body types. Spearman correlation analysis was performed between the total concentrations of PFAS in the muscle of wild fish of WYR and fish length, width, and weight (Fig. 5b). Specifically, the concentrations of most long-chain PFAS in fish muscle showed significant positive correlations (*p* < 0.05) with fish length, width, and weight. However, short-chain PFAS, PFOA, PFNA, and PFTeDA did not show this trend. PFOA only showed a significant correlation (*p* < 0.05) with fish weight. Pan et al. (2014b) found the concentrations of PFOS and PFUnDA in the muscle of the wild fish from Pearl River Delta region, South China, had significant positive correlations with their length and weight. The bioaccumulation of PFAS in fish relative to weight and length may be attributed to the fact that larger fish consume more food, occupy higher trophic levels and possess different dietary habits, which facilitate trophic transfer and biomagnification of PFAS along the food chain.

#### Bioaccumulation factors of PFAS in fish

Based on the concentrations of PFAS in water and fish muscle, the bioaccumulation factors (BAF) in six common fish were calculated by the equation (Text S4), as shown in Table S19 and Fig. 5c. Among the detected PFAS, the log BAF values of short-chain PFAS were below 3, whereas most of the long-chain PFAS demonstrated higher log BAF values (up to 5.15), except for PFNA. According to the study by Wilkinson et al. (2018), the log BAF values ranging from 3 to 3.7 indicated that the substances may have potential bioaccumulation, while the log BAF exceeding 3.7 suggested bio-accumulative behavior. Accordingly, most long-chain PFAS exhibited potential bioaccumulation in this study.

The polynomial regression analysis showed that log BAF and the carbon chain lengths of two categories (PFCA and PFSA) exhibited a strong polynomial fit for both categories of substances (PFCA: R^2^ = 0.8733, PFSA: R^2^ = 0.9125) (Fig. 5d). Similarly, PFAS bioaccumulation in fish were found to increase with carbon chain length in Pearl Bay in the study of Pan et al. (2014b). Additionally, the log BAF of PFSA was typically greater than that for PFCA. This observation is consistent with the findings of Dai et al. (2025), which examined marine organisms in the Arctic Valbat Island and indicated that sulfonic acid functional groups have a greater tendency to accumulate in biological organisms than carboxylic acids with the same chain length. Under the same conditions, the compounds possessing longer carbon chain length and containing sulfonic acid functional groups exhibited prolonged half-lives, higher protein water partition coefficients, demonstrated elevated uptake efficiency, and lower purification rates (Kelly et al., 2009; Martin et al., 2003; Sun et al., 2022). Spearman correlation analysis also revealed significant relationships (*p* < 0.05) between the total concentration of PFAS in wild fish and those in water and sediment (Fig. S4), suggesting that the ambient concentrations were also important factors in the bioaccumulation of wild fish in WYR.

Overall, the fish bioaccumulation of PFAS was affected by environmental exposure PFAS levels and the intrinsic characteristics of the PFAS (carbon chain length and functional groups). In addition, differences in feeding behavior and digestion rates among fish species may also influence bioaccumulation.

### Risk assessment in the aquatic environment

#### Ecological risk assessment in water

This study applied the species sensitivity distribution (SSD) method to assess the potential ecological risk associated with the exposure of aquatic organisms to PFAS in surface water. Acute toxicity data were derived from 240 standardized toxicity tests (including LC50 and EC50) encompassing 38 different aquatic species. The hazardous concentration for 5% of species (HC5) values were summarized in Table S8, except for PFBS, PFHxS, PFUnDA, PFDS, PFTrDA, PFTeDA (insufficient toxicity data). The calculated PNEC of most PFAS were lower than those calculated by Yu et al. (2025) using SSD, which may be attributed to differences in the species used for model construction. Based on the derived PNEC, ecological risks were evaluated using the RQ method. The calculated RQ values ranged from 2.10 × 10^−7^ to 7.94 × 10^−3^ (Fig. S5), indicating generally minimal risk for most PFAS. Among them, PFHpA, PFOA and PFOS showed relatively higher RQ values, with PFHpA (9.70 × 10^−4^–7.94 × 10^−3^), PFOA (7.46 × 10^−4^–1.87 × 10^−3^) and PFOS (4.15 × 10^−4^–3.88 × 10^−3^). These suggested that these compounds may warrant closer attention in the aquatic environment.

To further interpret the ecological implications from a regulatory perspective, measured PFAS concentrations were compared with the 2024 United States Environmental Protection Agency (USEPA) chronic water column criteria established for the protection of aquatic life (PFOA: 100000 ng/L, PFOS: 250 ng/L) (U.S. Environmental Protection Agency 2024). The measured concentrations of PFOA and PFOS at all sampling sites were within the USEPA chronic criterion.

Overall, the PNEC and RQ results indicated that 9 PFAS in the surface water of the WYR posed minimal ecological risks. Nevertheless, the relatively higher RQ of PFHpA, PFOA and PFOS observed at certain sites highlight the need for continued monitoring and management.

#### Health risk assessment in wild fish

The results of PFAS exposure risk assessment and average daily intake (ADI) in wild fish were shown in Table S14 and Table 2. A HR (hazard ratio) greater than 1 indicates that the acceptable exposure of pollutants exceeds the corresponding reference value and may suggest potential health concern (Chen et al., 2021a, 2021b). In this study, the HR values for all detected PFAS in fish from WYR were less than 1, suggesting relatively low health risk with consumption of the muscle tissue of the six fish species analyzed. Among the detected PFAS, the long-chain compounds (PFOA, PFOS, PFUnDA, and PFTrDA) showed higher HR values than the other PFAS, especially PFOS. This pattern suggested that long-chain PFAS may contribute more to dietary exposure through fish consumption.

**Table 2:** Average daily intake (ADI) (ng/kg/d) for local population under PFAS exposure through fish consumption from the WYR

Fish species	PFOA	PFDA	PFOS	PFUnDA	PFTrDA	PFTeDA
CI	0.17	0.084	0.48	0.067	0.17	0.047
CA	0.60	0.093	1.68	0.46	0.36	0.16
HM	0.33	0.15	1.38	0.31	0.69	0.12
CC	0.59	0.084	1.69	0.44	0.55	0.068
PP	0.27	0.24	0.75	0.54	0.36	0.035
CAB	0.58	0.11	1.12	0.17	0.014	0.091

CI: *Ctenopharyngodon idellus*, CA: *Carassius auratus*, HM: *Hypophthalmichthys molitrix*, CC: *Cyprinus carpio*, PP: *Parabramis pekinensis*, CAB: *Culter alburnus Basilews*

It should be noted that the reference values used in PFAS health risk assessment vary substantially among different studies and regulatory agencies, which introduces uncertainty into the evaluation. For example, the European Food Safety Authority (EFSA) stated that the tolerable intake for the sum of PFOA, PFNA, PFHxS and PFOS was 4.4 ng/kg/week (EFSA CONTAM Panel, 2020). This value was lower than the total ADI of four PFAS in this study, which ranged from 4.66 ng/kg/week for *Ctenopharyngodon idellus* to 16.10 ng/kg/week for *Carassius auratus*. In contrast, the reference values proposed for the Chinese population based on domestic epidemiological studies (Zhang et al., 2023) were 1.56 ng/kg/d for PFOA and 1.52 ng/kg/d for PFOS. These values were higher than the ADI in this study, except for PFOS for *Carassius auratus* (1.68 ng/kg/d) and *Cyprinus carpio* (1.69 ng/kg/d). Such differences likely reflect variations in exposure assumptions and population-specific parameters (e.g., daily fish intake, average body weight).

Overall, the present results suggested that consumption of wild fish from WYR was associated with relatively low health risk under TCEQ reference values, although the EFSA-based assessment indicated potential concern, and PFOS also exceeded the Chinese reference value for some fish species. Additionally, it is essential to acknowledge that the selective consumption of specific fish organs may influence PFAS exposure risk, as numerous studies have demonstrated elevated pollution of PFAS in fish livers and kidneys relative to muscle tissue (Brown et al., 2023; Fang et al., 2014; Pan et al., 2014b). Future research could enhance this analysis by concentrating on dietary intake surveys and the specific food types consumed by populations in different regions. Continuous monitoring, especially for long-chain PFAS, is essential to mitigate potential exposure risks.

## Conclusion

This study investigated the concentrations of 15 PFAS in water, sediment, and fish from 15 sampling points in the WYR. Overall, PFAS in surface water were mainly short-chain compounds, with PFBS being the most abundant (34.0%). PFAS in sediment were mainly long-chain compounds, with PFOS being the dominant compound (64.0%). PFOS was also the most prevalent compound in wild fish, accounting for 42.2%. The majority of long-chain PFAS demonstrated bioaccumulation capacity in wild fish from WYR. The annual PFAS flux from WYR to the Yangtze River was 2.72t. The Unmix model revealed the predominant sources of PFAS in WYR: metal plating (41%) of the total sources, followed by electronic manufacturing/food package (30%) and textile processing (29%). Risk assessment in water indicated that the detected PFAS posed minimal risk based on the PNEC and RQ results. Health risk assessment based on the 15 target PFAS analysis in fish muscle suggested relatively low risk under TCEQ reference values. However, when evaluated against EFSA standards, the results suggested potential concern for all fish species except for CI. The findings of this study contribute valuable insights into PFAS contamination in water, sediment and fish, as well as their sources, fluxes and the potential risks in WYR and provide useful support for decision-makers in formulating appropriate control measures.

## Supplementary Information

Below is the link to the electronic supplementary material.

Supplementary file1 (DOCX 1023 KB)

## Data Availability

No datasets were generated or analysed during the current study.
