# Supplementary material for: From industry to consumer use: unraveling PFAS sources, bioaccumulation, and fluxes in a year-long catchment monitoring study
Source: Environ Geochem Health. 2026 Apr 30;48(7):325. doi: 10.1007/s10653-026-03200-0 (PMC13133208; doi:10.1007/s10653-026-03200-0)
Supplement: Supplementary file 1 — Supplementary file1 (DOCX 1023 KB) [file 10653_2026_3200_MOESM1_ESM.docx]

**Supplementary material**

**From Plating to Plates: Unraveling PFAS Sources, Bioaccumulation, and Fluxes in a year-long catchment monitoring study**

Yuheng Chen^a, b, 1^, Shanqi Zhou ^b, 1^, Tianyi Li^a^, Yulin Chen^a^, Zulin Zhang^a, c,*^

*^a^* *Hubei Key Laboratory of Mineral Resources Processing and Environment, School of Resources and Environmental Engineering,* *Wuhan University of Technology, Wuhan 430070, China*

*^b^Xianghu Laboratory, Hangzhou 311231, China*

*^c^The James Hutton Institute, Aberdeen AB15 8QH, UK*

^1^ These authors contributed equally to the present work

* Corresponding author：[zulin.zhang@hutton.ac.uk](mailto:zulin.zhang@hutton.ac.uk) (Zulin Zhang)

**Text S1** Sample collection.

**Text S2** Sediment samples extraction.

**Text S3** Effects of seasonal variation on PFAS in water and sediment.

**Text S4** Bioaccumulation calculation.

**Table S1** The analyte formula, acronym, and optimum LC-MS/MS parameters for multiple reaction monitoring (MRM) acquisition conditions of individual PFAS.

**Table S2** Basic information for each sampling site of the Wuhan section of Yangtze River.

**Table S3** Basic information for fish sample species and sampling site of the Wuhan section of Yangtze River.

**Table S4** Gradient elution procedure for LC-MS/MS.

**Table S5** Recovery and LOQ of target compounds in water and sediments by LC-MS/MS.

**Table S6** Concentration (ng/L) of PFAS in water at S1, S8, S15 within the Wuhan section of the Yangtze River.

**Table S7** Summary of the flow rates in Wuhan section of the Yangtze River.

**Table S8** Summary of PNEC values for PFAS.

**Table S9** Reference doses (RfD) of PFAS.

**Table S10** Concentrations (ng/L) of PFAS detected in the water of the Wuhan section of Yangtze River.

**Table S11** Concentrations (ng/g, dw) of PFAS detected in the sediments of the Wuhan section of Yangtze River.

**Table S12** Concentrations (ng/g, dw) of PFAS detected in wild fish of the Wuhan section of Yangtze River.

**Table S13** Standardized distribution coefficients log Koc (L/kg) for PFAS in the Wuhan section of the Yangtze River.

**Table S14** Exposure risk assessment of PFAS in wild fish in the Wuhan section of Yangtze River.

**Table S15** Concentrations (ng/L) and detection frequencies of PFAS in water of the Wuhan section of the Yangtze River in summer (n=15) and winter (n=15).

**Table S16** Concentrations (ng/g, dw) and detection frequencies of PFAS in sediments of the Wuhan section of the Yangtze River in summer (n=15) and winter (n=15).

**Table S17** The average daily fluxes (kg/d) in different sites and seasons.

**Table S18** The annual fluxes (t/a) in different sites.

**Table S19** Bioaccumulation Factor (log BAF) of six common fish species in the Wuhan section of Yangtze River.

**Fig. S1.** The fitting curve predicted by Unmix model.

**Fig. S2.** The results of Spearman’s correlation of PFAS and TOC in sediment in summer (a), and in winter (b).

**Fig. S3.** The correlation between the log KOC of several substance and concentrations in sediment and water, TOC in sediment.

**Fig. S4.** The correlation of the total concentration of 15 PFAS (c) and PFOS (d) in different media..

**Fig. S5.** Ecological risk in water of WYR.

**Text S1 Sample collection**

A total of 18 fish samples from WYR were sourced from anglers during the sampling period (Table S3). Surface water samples (500 mL) were stored in polypropylene bottles which had been prewashed by distilled water and methanol. Sediment samples were acquired from the top 0-10 cm layer utilizing a stainless-steel grab and carefully wrapped in aluminum foil.

**Text S2 Samples extraction**

The collected water samples were filtered through the 0.7 μm glass fiber membrane on the same day, 500 ml of filtered water samples were collected and spiked with 5 ng internal standard. Subsequently, the SPE column was used to complete the extraction process. The SPE column was activated with 10 ml of chromatographic methanol and 10 ml of water for HPLC analysis, and then absorbed the water samples under negative pressure. When the target compounds had been enriched on the SPE column, the column was drenched with 10 ml of water, dried in vacuum for 30 min, and eluted with 10 ml of chromatographic methanol. The eluate was concentrated under nitrogen at 35 °C to near dryness, and then re-dissolved in 1 ml of 1:1 methanol in water, passed through the 0.22 μm nylon filter membrane. Finally, it was loaded into a 1.5 ml injection vial and stored at 4 °C.

5 g sediment sample was weighed into a 50 mL polypropylene (PP) centrifuge tube and 5 ng mixed internal standards were added. Then 2 mL cleaned Milli-Q water was added for moistening and the mixture was vortexed for 1 min. Subsequently, 2 mL of 0.25 M sodium carbonate buffer and 1 mL of 0.5 MTBAHS were added to the centrifuge tube and sonicated for 20 min at 60 °C. After that, 5 mL of MTBE was added and shaken for 20 min. After centrifuging for 10 min at 8000 rpm, the supernatant was collected. This process was repeated one more time to ensure that the target compounds were extracted. The extracts were evaporated to dryness with a gentle stream of nitrogen and reconstituted in 1 mL 1:1 methanol water into a 1.5 mL centrifuge tube. Subsequently, the tubes were centrifuged at 12,000 rpm for 5 min. The supernatant solution was filtered through a 0.22 µm nylon filter, and then transferred into PP vials and analyzed with LC-MS/MS.

A precisely weighed 0.5 g aliquot of fish sample was transferred into a 50 mL polypropylene centrifuge tube and spiked with 5 ng of a mixed internal standard. The mixture was vortexed for 1 min, after which 10 mL of 10 mmol/L KOH in methanol was added to the centrifuge tube and shaken for 16 h at 180 rpm. After centrifuging at 8000 rpm for 10 min, the supernatant was carefully decanted into a polypropylene bottle. To reduce the methanol concentration to less than 5%, 300 mL of HPLC-grade water was added to the bottle. The subsequent solid-phase extraction procedure was analogous to that employed for water samples.

For water, sediment and fish samples, the eluate was concentrated under nitrogen at 35 °C to near dryness, and then re-dissolved in 1 ml of 1:1 methanol in water, passed through the 0.22 μm nylon filter membrane. Finally, it was loaded into a 1.5 ml injection vial and stored at 4 °C.

**Text S3 Effects of seasonal variation on PFAS in water and sediment**

In order to further explore the influence of seasonal variation on PFAS in water and sediment, the distribution coefficient (K_d_) and standardized distribution coefficient (K_oc_) were introduced to characterize the migration ability of PFAS between the two phases. The partition coefficient can be obtained by the quotient of PFAS concentration (C_s_) in sediment and PFAS concentration (C_w_) in water, as shown in formula (Eq. 1). According to the organic matter content (f_oc_) of sediment samples in each sampling point, the distribution coefficient was further normalized to obtain the standardized distribution coefficient, as shown in formula (Eq. 2).

$$\begin{aligned} K_{d}=\frac{C_{s}}{C_{w}}\#\left（ 1 \right） \end{aligned}$$

$$\begin{aligned} K_{\mathrm{oc}}=\frac{K_{d}}{f_{\mathrm{oc}}}\#\left（ 2 \right） \end{aligned}$$

**Text S4 Bioaccumulation calculation**

Bioaccumulation factor (BAF) refers to the ratio of the accumulated concentration of pollutants in the organism. It caused by exposure (excluding ingestion) to the equilibrium concentration of such pollutants in water^[2]^, calculate by formula (Eq. 3).

$$\begin{aligned} BAF=\frac{C_{f}}{C_{w}}\#\left( 3 \right) \end{aligned}$$

Where C_w_ is the concentration of the target pollutant in water, C_f_ is the concentration of the target pollutant in the tissue of the corresponding biological sample.

**Table S1** The analyte formula, acronym, and optimum LC-MS/MS parameters for multiple reaction monitoring (MRM) acquisition conditions of individual PFAS.

| Compound | Ion Transition (m/z) | Entrance Voltage | Collision Cell Lens 2 | Collision Energy | Corresponding Internal Standard |
| --- | --- | --- | --- | --- | --- |
| Perfluorobutanoic acid (PFBA) | 213>169* | -1 | 37 | 10 | MPFBA |
| Perfluoropentanoic acid (PFPeA) | 263>219* | 0 | 44 | 10 | MPFHxA |
|  | 263>19.2 | 0 | 44 | 20 |  |
| Perfluorohexanoic acid (PFHxA) | 313.1>269* | -1 | 48 | 13 | MPFHxA |
|  | 313.1>119.2 | -1 | 76 | 34 |  |
| Perfluoroheptanoic acid (PFHpA) | 363.1>319* | -8 | 85 | 13 | MPFOA |
|  | 363.1>169 | -12 | 92 | 25 |  |
| Perfluorooctanoic acid (PFOA) | 412.8>368.8* | -14 | 68 | 15 | MPFOA |
|  | 412.8>168.8 | 0 | 72 | 26 |  |
| Perfluorononanoic acid (PFNA) | 463.2>418.9* | -9 | 100 | 15 | MPFNA |
|  | 463.2>218.9 | -12 | 92 | 21 |  |
| Perfluorodecanoic acid (PFDA) | 513>469* | 0 | 89 | 15 | MPFDA |
|  | 513>219.2 | -16 | 124 | 24 |  |
| Perfluoroundecanoic acid (PFUnDA) | 563.1>519* | -7 | 100 | 16 | MPFUnDA |
|  | 563.1>169.2 | -13 | 92 | 33 |  |
| Perfluorododecanoic acid (PFDoDA) | 613.1>569* | -2 | 95 | 15 | MPFDoDA |
| Perfluorotridecanoic acid (PFTrDA) | 663>619* | -6 | 136 | 18 | MPFDoDA |
|  | 663>169.2 | -10 | 117 | 3 |  |
| Perfluorotetradecanoic acid (PFTeDA) | 713>668.9* | -20 | 156 | 23 | MPFDoDA |
|  | 713>168.9 | -23 | 127 | 48 |  |
| Perfluoro-1-butanesulfonic acid (PFBS) | 299>80.1* | -20 | 104 | 75 | MPFHxS |
|  | 299>98.9 | -1 | 76 | 49 |  |
| Perfluoro-1-hexanesulfonic acid (PFHxS) | 399>80* | -52 | 76 | 55 | MPFHxS |
|  | 399>99 | -35 | 155 | 45 |  |
| Perfluoro-1-octanesulfonic acid (PFOS) | 500>80* | -55 | 156 | 99 | MPFOS |
|  | 500>99.1 | -60 | 112 | 57 |  |
| Perfluoro-1-decanesulfonic acid (PFDS) | 599.1>80* | -64 | 172 | 125 | MPFOS |
|  | 599.1>99 | -50 | 216 | 75 |  |
| Perfluoro-n-[^13^C_4_]butanoic acid (MPFBA) | 217.1>172* | -1 | 20 | 10 |  |
| Perfluoro-n-[1,2-^13^C_2_]hexanoic acid (MPFHxA) | 315.2>270* | -2 | 44 | 13 |  |
| Perfluoro-n-[1,2,3,4-^13^C_4_]octanoic acid (MPFOA) | 417.2>372* | -11 | 60 | 15 |  |
| Perfluoro-n-[1,2,3,4,5-^13^C_5_]nonanoic acid (MPFNA) | 468.2>423* | -5 | 89 | 16 |  |
| Perfluoro-n-[1,2-^13^C_2_]decanoic acid (MPFDA) | 515.2>470* | -24 | 112 | 19 |  |
| Perfluoro-n-[1,2-^13^C_2_]undecanoic acid (MPFUnDA) | 565.1>520* | -1 | 96 | 16 |  |
| Perfluoro-n-[1,2-^13^C_2_]dodecanoic acid (MPFDoDA) | 615.2>570* | -14 | 110 | 18 |  |
| Sodium perfluoro-1-[^18^O_2_]hexanesulfonic acid (MPFHxS) | 403.2>84* | -47 | 120 | 77 |  |
| Sodium perfluoro-1-[1,2,3,4-^13^C_4_]octanesulfonic acid (MPFOS) | 503.2>80* | -46 | 156 | 88 |  |

* The product ion used for quantification

**Table S2** Basic information for each sampling site of the Wuhan section of Yangtze River.

| Sampling site | Geographic location | | City\County | Province | Land ues |
| --- | --- | --- | --- | --- | --- |
|  | (E, N) | |  |  |  |
| S1 | 114°05'22'' | 30°06'10'' | Xianning | Hubei | Agricultural area |
| S2 | 113°52'41'' | 30°16'31'' | Xianning | Hubei | Agricultural area |
| S3 | 114°02'51'' | 30°12'53'' | Wuhan | Hubei | Agricultural area |
| S4 | 114°07'29'' | 30°20'14'' | Wuhan | Hubei | Urban area/ Argricultural area |
| S5 | 114°11'23'' | 30°25'32'' | Wuhan | Hubei | Urban area/ Argricultural area |
| S6 | 114°14'38'' | 30°30'29'' | Wuhan | Hubei | Urban area |
| S7 | 114°16'05'' | 30°32'10'' | Wuhan | Hubei | Urban area |
| S8 | 114°18'31'' | 30°36'01'' | Wuhan | Hubei | Urban area |
| S9 | 114°25'09'' | 30°39'29'' | Wuhan | Hubei | Urban area/ Argricultural area |
| S10 | 114°30'42'' | 30°41'23'' | Wuhan | Hubei | Agricultural area/ Industrial area |
| S11 | 114°34'43'' | 30°36'09'' | Wuhan | Hubei | Industrial area/ Agricultural area |
| S12 | 114°39'39'' | 30°34'49'' | Wuhan | Hubei | Agricultural area/ Urban area |
| S13 | 114°44'16'' | 30°36'32'' | Huanggang | Hubei | Agricultural area |
| S14 | 114°50'19'' | 30°32'07'' | Huanggang | Hubei | Agricultural area |
| S15 | 114°54'23'' | 30°25'02'' | Huanggang | Hubei | Industrial area |

**Table S3** Basic information for fish sample species and sampling site of the Wuhan section of Yangtze River.

| Species | Name | Sampling time | Sampling site |
| --- | --- | --- | --- |
| *Ctenopharyngodon idellus* | CI1 | 2022-7-19 | S8 |
|  | CI2 |  |  |
|  | CI3 | 2022-7-3 | S5 |
|  | CI4 |  |  |
|  | CI5 |  |  |
|  | CI6 |  |  |
|  | CI7 |  |  |
|  | CI8 |  |  |
|  | CI9 |  |  |
| *Carassius auratus* | CA1 | 2022-8-4 | S14 |
|  | CA2 |  |  |
|  | CA3 |  |  |
| *Hypophthalmichthys molitrix* | HM1 | 2022-7-19 | S6 |
|  | HM2 |  |  |
|  | HM3 |  |  |
| *Cyprinus carpio* | CC1 | 2022-7-19 | S9 |
| *Parabramis pekinensis* | PP1 | 2022-7-19 | S9 |
| *Culter alburnus Basilewsky* | CAB1 | 2022-7-19 | S8 |

**Table S4** Gradient elution procedure for LC-MS/MS.

| Time (min) | Mobile phase^1^ | | Flow (ml/min) |
| --- | --- | --- | --- |
|  | A (%) | B (%) |  |
| 0 | 95 | 5 | 0.3 |
| 1 | 95 | 5 | 0.3 |
| 3 | 70 | 30 | 0.3 |
| 13 | 5 | 95 | 0.3 |
| 15 | 5 | 95 | 0.3 |
| 16 | 95 | 5 | 0.3 |
| 20 | 95 | 5 | 0.3 |

**Table S5** Recovery and LOQ of target compounds in water and sediments by LC-MS/MS.

| Compound | Sediment | | | Water | | | Fish | | |
| --- | --- | --- | --- | --- | --- | --- | --- | --- | --- |
|  | Recovery±  RSD (%) | LOD (ng/g) | LOQ (ng/g) | Recovery (%) | LOD (ng/L) | LOQ (ng/L) | Recovery (%) | LOD (ng/g) | LOQ (ng/g) |
| PFBA | 109.8 ± 7.10 | 0.001 | 0.002 | 112.2 ± 4.32 | 0.010 | 0.020 | 85.0 ± 3.32 | 0.001 | 0.002 |
| PFPeA | 98.56 ± 9.44 | 0.005 | 0.015 | 90.28 ± 7.53 | 0.050 | 0.150 | 84.7 ± 8.34 | 0.005 | 0.015 |
| PFHxA | 113.7 ± 4.66 | 0.005 | 0.018 | 115.2 ± 2.16 | 0.050 | 0.180 | 91.6 ± 9.76 | 0.005 | 0.018 |
| PFHpA | 91.54 ± 1.46 | 0.004 | 0.012 | 114.2 ± 6.41 | 0.040 | 0.120 | 91.2 ± 5.72 | 0.004 | 0.012 |
| PFOA | 111.6 ± 5.43 | 0.001 | 0.003 | 104.8 ± 1.33 | 0.010 | 0.030 | 88.8 ± 8.12 | 0.001 | 0.003 |
| PFNA | 109.6 ± 8.45 | 0.003 | 0.010 | 98.17 ± 3.45 | 0.030 | 0.100 | 84.3 ± 5.29 | 0.003 | 0.010 |
| PFDA | 105.8 ± 7.66 | 0.003 | 0.010 | 108.6 ± 5.21 | 0.030 | 0.100 | 93.8 ± 7.53 | 0.003 | 0.01 |
| PFUnDA | 92.02 ± 9.01 | 0.005 | 0.015 | 99.41 ± 3.74 | 0.050 | 0.150 | 101.0 ± 3.72 | 0.005 | 0.015 |
| PFDoA | 87.30 ± 1.72 | 0.009 | 0.030 | 101.5 ± 5.62 | 0.090 | 0.300 | 105.0 ± 9.51 | 0.009 | 0.030 |
| PFTrDA | 62.30 ± 5.02 | 0.009 | 0.030 | 103.0 ± 2.54 | 0.090 | 0.300 | 69.9 ± 6.37 | 0.009 | 0.030 |
| PFTeDA | 49.93 ± 6.44 | 0.018 | 0.060 | 93.0 ± 8.32 | 0.180 | 0.600 | 60.2 ± 4.25 | 0.018 | 0.060 |
| PFBS | 97.17 ± 1.35 | 0.001 | 0.002 | 110.6 ± 3.21 | 0.010 | 0.020 | 90.0 ± 3.77 | 0.001 | 0.002 |
| PFHxS | 110.5 ± 3.12 | 0.001 | 0.002 | 119.4 ± 4.33 | 0.010 | 0.020 | 88.30 ± 5.41 | 0.001 | 0.002 |
| PFOS | 113.4 ± 7.34 | 0.001 | 0.002 | 118.8 ± 7.21 | 0.010 | 0.020 | 97.6 ± 8.09 | 0.001 | 0.002 |
| PFDS | 52.17 ± 4.19 | 0.004 | 0.012 | 85.31 ± 6.43 | 0.040 | 0.120 | 62.9 ± 4.7 3 | 0.004 | 0.012 |

**Table S6** Concentration (ng/L) of PFAS in water at S1, S8, S15 within the Wuhan section of the Yangtze River.

| **TIME** | **SITE** | **PFBA** | **PFPeA** | **PFHxA** | **PFBS** | **PFHpA** | **PFOA** | **PFHxS** | **PFNA** | **PFOS** | **PFDS** |
| --- | --- | --- | --- | --- | --- | --- | --- | --- | --- | --- | --- |
| 2022.6 | S1 | 0.72 | 0.26 | 0.46 | 0.36 | 0.24 | 1.34 | 0.014 | 0.14 | 0.27 | <LOD |
| 2022.6 | S8 | 0.84 | 0.25 | 0.44 | 0.68 | 0.64 | 1.41 | 0.057 | 0.17 | 0.50 | <LOD |
| 2022.6 | S15 | 1.02 | 0.25 | 0.55 | 1.44 | 0.83 | 1.82 | 0.079 | 0.21 | 0.29 | <LOD |
| 2022.7 | S1 | 1.00 | 0.42 | 0.59 | 1.35 | 0.12 | 2.55 | <LOD | 0.18 | 0.26 | 0.126 |
| 2022.7 | S8 | 1.34 | 1.89 | 0.69 | 1.02 | 0.90 | 1.51 | 0.043 | 0.21 | 0.46 | <LOD |
| 2022.7 | S15 | <LOD | <LOD | 0.66 | 2.35 | 0.37 | 1.99 | 0.057 | 0.28 | 0.28 | <LOD |
| 2022.9 | S1 | <LOD | 0.91 | 1.39 | 0.99 | 0.30 | 1.26 | 0.043 | 0.14 | 0.23 | 0.065 |
| 2022.9 | S8 | 3.58 | 1.37 | 1.25 | 1.02 | 0.33 | 1.55 | 0.078 | 0.22 | 0.55 | 0.049 |
| 2022.9 | S15 | 1.38 | 1.73 | 1.99 | 1.70 | 0.19 | 1.74 | 0.10 | 0.21 | 0.34 | <LOD |
| 2022.10 | S1 | 1.15 | 1.00 | 1.73 | 3.14 | 0.38 | 2.15 | 0.077 | 0.29 | 0.38 | <LOD |
| 2022.10 | S8 | 1.21 | 0.60 | 1.53 | 1.76 | 0.42 | 2.22 | 0.067 | 0.34 | 0.47 | <LOD |
| 2022.10 | S15 | 2.35 | 2.00 | 1.85 | 1.77 | 0.43 | 2.35 | 0.14 | 0.23 | 0.33 | <LOD |
| 2022.12 | S1 | <LOD | 1.22 | 2.00 | 2.04 | 0.32 | 1.96 | 0.14 | 0.19 | 0.41 | <LOD |
| 2022.12 | S8 | 3.29 | 0.66 | 2.09 | 1.87 | 0.35 | 2.26 | 0.10 | 0.30 | 0.43 | <LOD |
| 2022.12 | S15 | 2.40 | 0.50 | 2.63 | 5.37 | 0.40 | 1.96 | 0.23 | 0.22 | 0.57 | <LOD |
| 2023.2 | S1 | 2.63 | 1.56 | 3.51 | 3.08 | 0.38 | 3.55 | 0.037 | 0.13 | 0.27 | <LOD |
| 2023.2 | S8 | 2.44 | 1.73 | 2.69 | 3.34 | 0.50 | 3.19 | 0.34 | 0.12 | 0.30 | <LOD |
| 2023.2 | S15 | 2.02 | 1.67 | 3.00 | 3.30 | 0.37 | 3.26 | 0.20 | 0.058 | 0.68 | <LOD |
| 2023.3 | S1 | <LOD | 2.00 | 2.79 | 13.06 | 0.52 | 3.60 | 0.069 | 0.35 | 0.45 | 0.068 |
| 2023.3 | S8 | <LOD | 4.13 | 1.50 | 26.50 | 0.42 | 2.36 | 0.78 | 0.33 | 0.52 | <LOD |
| 2023.3 | S15 | 1.51 | 1.78 | 4.43 | 27.35 | 0.57 | 4.88 | 0.37 | 0.25 | 0.61 | <LOD |
| 2023.4 | S1 | <LOD | 0.48 | 3.36 | 2.65 | 0.65 | 2.24 | 0.050 | 0.36 | 0.064 | <LOD |
| 2023.4 | S8 | <LOD | 1.16 | 3.46 | 3.56 | 0.66 | 2.77 | 0.15 | 0.59 | 0.64 | <LOD |
| 2023.4 | S15 | <LOD | 1.05 | 4.19 | 4.88 | 0.63 | 2.67 | 0.28 | 0.35 | 0.21 | <LOD |

**Table S7** Summary of the flow rates in Wuhan section of the Yangtze River.

| Q (m^3^/s) | S1 | S2 | S3 |
| --- | --- | --- | --- |
| 2022.06 | 38075 | 40710 | 41074 |
| 2022.07 | 30310 | 30935 | 31205 |
| 2022.08 | 16434 | 17139 | 17274 |
| 2022.09 | 9825 | 10596 | 10668 |
| 2022.10 | 9864 | 10361 | 10431 |
| 2022.11 | 8536 | 8777 | 8831 |
| 2022.12 | 8686 | 9165 | 9223 |
| 2023.01 | 8553 | 8627 | 8680 |
| 2023.02 | 9059 | 9409 | 9469 |
| 2023.03 | 9264 | 9504 | 9566 |
| 2023.04 | 13575 | 14373 | 14482 |
| 2023.05 | 16551 | 17613 | 17753 |

*The flux of S8 was from the average daily flow of Hankou Hydrological Station, and the data of S1 and S15 were fitted by the daily flow data of Luoshan, Hankou, Jiujiang Station.

**Table S8** Summary of HC5 and PNEC values for PFAS.

| Target PFAS | HC5 (μg/L) | PNEC (μg/L) |
| --- | --- | --- |
| PFBA | 119.0 | 23.80 |
| PFPeA | 38.70 | 7.74 |
| PFHxA | 92.95 | 18.59 |
| PFHpA | 0.61 | 0.12 |
| PFOA | 10.10 | 2.02 |
| PFNA | 1.37 | 0.27 |
| PFDA | 1.38 | 0.28 |
| PFOS | 1.95 | 0.39 |
| PFDoA | 18.74 | 3.75 |

**Table S9** Reference doses (RfD) of PFAS.

| Compound | RfD (ng/kg/d) |
| --- | --- |
| PFBA | 1000 |
| PFPeA | 500 |
| PFHxA | 500 |
| PFBS | 1400 |
| PFHpA | 23 |
| PFOA | 12 |
| PFHxS | 3.8 |
| PFNA | 12 |
| PFDA | 15 |
| PFOS | 23 |
| PFUnDA | 12 |
| PFDoA | 12 |
| PFDS | 12 |
| PFTrDA | 12 |
| PFTeDA | 12 |

*From Texas Commission on Environmental Quality.

**Table S10** Concentrations (ng/L) of PFAS detected in the water of the Wuhan section of Yangtze River.

| Time | Site | PFBA | PFPeA | PFHxA | PFBS | PFHpA | PFOA | PFHxS | PFNA | PFDA | PFOS | PFUnDA | PFDoA | PFDS | PFTrDA | PFTeDA |
| --- | --- | --- | --- | --- | --- | --- | --- | --- | --- | --- | --- | --- | --- | --- | --- | --- |
| July | S1 | 1.00 | 0.42 | 0.59 | 1.35 | 0.12 | 2.55 | < LOD | 0.18 | < LOD | 0.26 | < LOD | < LOD | 0.13 | < LOD | < LOD |
|  | S2 | < LOD | 1.23 | 0.97 | 1.17 | 0.15 | 2.06 | < LOD | 0.24 | < LOD | 0.16 | < LOD | < LOD | < LOD | < LOD | < LOD |
|  | S3 | < LOD | 0.96 | 0.71 | 2.02 | 0.19 | 1.90 | < LOD | 0.26 | < LOD | 0.19 | < LOD | < LOD | < LOD | < LOD | < LOD |
|  | S4 | < LOD | 0.88 | 0.79 | 1.06 | 0.34 | 1.65 | 0.033 | 0.13 | < LOD | 0.21 | < LOD | < LOD | < LOD | < LOD | < LOD |
|  | S5 | 1.62 | 0.79 | 0.75 | 1.06 | 0.35 | 1.66 | 0.008 | 0.15 | < LOD | 0.21 | < LOD | < LOD | < LOD | < LOD | < LOD |
|  | S6 | 1.56 | 1.75 | 0.95 | 1.39 | 0.71 | 2.68 | 0.071 | 0.23 | < LOD | 0.98 | < LOD | < LOD | < LOD | < LOD | < LOD |
|  | S7 | 1.32 | 1.70 | 0.71 | 1.18 | 0.27 | 1.90 | 0.034 | 0.26 | < LOD | 0.70 | < LOD | < LOD | < LOD | < LOD | < LOD |
|  | S8 | 1.34 | 1.89 | 0.69 | 1.02 | 0.90 | 1.51 | 0.043 | 0.21 | < LOD | 0.46 | < LOD | < LOD | < LOD | < LOD | < LOD |
|  | S9 | 1.29 | 1.16 | 0.83 | 0.92 | 0.30 | 1.91 | 0.032 | 0.15 | < LOD | 0.30 | < LOD | < LOD | < LOD | < LOD | < LOD |
|  | S10 | < LOD | < LOD | 1.29 | 35.50 | 0.97 | 2.82 | 0.83 | 0.30 | < LOD | 1.51 | < LOD | < LOD | < LOD | < LOD | < LOD |
|  | S11 | < LOD | 0.28 | 0.69 | 4.94 | 0.40 | 1.74 | 0.10 | 0.18 | < LOD | 0.39 | < LOD | < LOD | < LOD | < LOD | < LOD |
|  | S12 | < LOD | < LOD | 0.74 | 4.82 | 0.45 | 2.28 | 0.069 | 0.10 | < LOD | 0.34 | < LOD | < LOD | < LOD | < LOD | < LOD |
|  | S13 | < LOD | < LOD | 0.81 | 4.81 | 0.49 | 1.81 | 0.078 | 0.16 | < LOD | 0.37 | < LOD | < LOD | < LOD | < LOD | < LOD |
|  | S14 | < LOD | 0.11 | 0.68 | 3.14 | 0.34 | 1.95 | 0.065 | 0.21 | < LOD | 0.32 | < LOD | < LOD | < LOD | < LOD | < LOD |
|  | S15 | < LOD | < LOD | 0.66 | 2.35 | 0.37 | 1.99 | 0.057 | 0.28 | < LOD | 0.28 | < LOD | < LOD | < LOD | < LOD | < LOD |
| December | S1 | < LOD | 1.22 | 2.00 | 2.04 | 0.32 | 1.96 | 0.14 | 0.19 | < LOD | 0.41 | < LOD | < LOD | < LOD | < LOD | < LOD |
|  | S2 | < LOD | 1.00 | 2.19 | 5.24 | 0.46 | 2.26 | 0.12 | 0.24 | < LOD | 0.23 | < LOD | < LOD | < LOD | < LOD | < LOD |
|  | S3 | 2.88 | 1.69 | 2.22 | 2.82 | 0.33 | 2.12 | 0.20 | 0.23 | < LOD | 0.97 | < LOD | < LOD | < LOD | < LOD | < LOD |
|  | S4 | 3.11 | 1.74 | 2.30 | 3.23 | 0.32 | 2.35 | 0.21 | 0.23 | < LOD | 0.21 | < LOD | < LOD | < LOD | < LOD | < LOD |
|  | S5 | 2.86 | 1.07 | 2.26 | 2.22 | 0.33 | 2.41 | 0.12 | 0.25 | < LOD | 0.29 | < LOD | < LOD | < LOD | < LOD | < LOD |
|  | S6 | < LOD | 0.65 | 2.32 | 1.91 | 0.47 | 2.62 | 0.11 | 0.18 | < LOD | 0.47 | < LOD | < LOD | < LOD | < LOD | < LOD |
|  | S7 | 2.29 | 0.70 | 2.50 | 1.97 | 0.54 | 2.50 | 0.20 | 0.23 | < LOD | 0.75 | < LOD | < LOD | < LOD | < LOD | < LOD |
|  | S8 | 3.29 | 0.66 | 2.09 | 1.87 | 0.35 | 2.26 | 0.10 | 0.30 | < LOD | 0.43 | < LOD | < LOD | < LOD | < LOD | < LOD |
|  | S9 | 3.39 | 1.17 | 2.36 | 3.58 | 0.30 | 2.68 | 0.17 | 0.20 | < LOD | 0.43 | < LOD | < LOD | < LOD | < LOD | < LOD |
|  | S10 | 3.20 | 0.77 | 1.84 | 3.54 | 0.45 | 2.54 | 0.48 | 0.32 | < LOD | 0.70 | < LOD | < LOD | < LOD | < LOD | < LOD |
|  | S11 | 3.83 | 0.56 | 2.03 | 2.36 | 0.35 | 2.16 | 0.20 | 0.29 | < LOD | 0.35 | < LOD | < LOD | < LOD | < LOD | < LOD |
|  | S12 | 12.56 | 0.45 | 2.09 | 4.60 | 0.36 | 1.89 | 0.16 | 0.23 | < LOD | 0.45 | < LOD | < LOD | < LOD | < LOD | < LOD |
|  | S13 | 2.73 | 0.73 | 2.20 | 2.21 | 0.55 | 3.79 | 0.14 | 0.30 | < LOD | 0.30 | < LOD | < LOD | < LOD | < LOD | < LOD |
|  | S14 | 6.27 | 0.64 | 2.29 | 2.51 | 0.38 | 2.22 | 0.17 | 0.24 | < LOD | 0.49 | < LOD | < LOD | < LOD | < LOD | < LOD |
|  | S15 | 2.40 | 0.50 | 2.63 | 5.37 | 0.40 | 1.96 | 0.23 | 0.22 | < LOD | 0.57 | < LOD | < LOD | < LOD | < LOD | < LOD |

Note, LOD: method limit of detection.

**Table S11** Concentrations (ng/g, dw) of PFAS detected in the sediments of the Wuhan section of Yangtze River.

| Time | Site | | PFBA | | PFPeA | | PFHxA | | PFBS | | PFHpA | | PFOA | | PFHxS | | PFNA | | PFDA | | PFOS | | PFUnDA | | PFDoA | | PFDS | | PFTrDA | | PFTeDA | |
| --- | --- | --- | --- | --- | --- | --- | --- | --- | --- | --- | --- | --- | --- | --- | --- | --- | --- | --- | --- | --- | --- | --- | --- | --- | --- | --- | --- | --- | --- | --- | --- | --- |
| July | | S1 | | < LOD | | < LOD | | < LOD | | 0.0025 | | < LOD | | < LOD | | < LOD | | < LOD | | < LOD | | 0.0046 | | < LOD | | < LOD | | < LOD | | < LOD | | < LOD |
|  |  | S2 | | < LOD | | < LOD | | < LOD | | 0.0032 | | < LOD | | 0.019 | | 0.0013 | | 0.021 | | < LOD | | 0.044 | | < LOD | | < LOD | | < LOD | | < LOD | | < LOD |
|  |  | S3 | | < LOD | | < LOD | | < LOD | | 0.0077 | | < LOD | | < LOD | | < LOD | | < LOD | | < LOD | | 0.011 | | < LOD | | < LOD | | < LOD | | < LOD | | < LOD |
|  |  | S4 | | < LOD | | < LOD | | < LOD | | 0.0097 | | < LOD | | 0.035 | | 0.014 | | 0.026 | | 0.040 | | 0.30 | | 0.053 | | < LOD | | 0.0073 | | < LOD | | < LOD |
|  |  | S5 | | 0.026 | | < LOD | | < LOD | | 0.0098 | | < LOD | | 0.0401 | | < LOD | | < LOD | | < LOD | | 0.050 | | < LOD | | < LOD | | < LOD | | < LOD | | < LOD |
|  |  | S6 | | < LOD | | < LOD | | < LOD | | 0.0062 | | < LOD | | < LOD | | < LOD | | < LOD | | < LOD | | 0.13 | | < LOD | | < LOD | | < LOD | | < LOD | | < LOD |
|  |  | S7 | | < LOD | | < LOD | | < LOD | | < LOD | | < LOD | | < LOD | | < LOD | | < LOD | | < LOD | | < LOD | | < LOD | | < LOD | | < LOD | | < LOD | | < LOD |
|  |  | S8 | | < LOD | | < LOD | | < LOD | | 0.0080 | | < LOD | | 0.025 | | 0.014 | | < LOD | | < LOD | | 0.047 | | < LOD | | < LOD | | < LOD | | < LOD | | < LOD |
|  |  | S9 | | < LOD | | < LOD | | 0.035 | | 0.0084 | | 0.050 | | 0.66 | | 0.041 | | < LOD | | < LOD | | 3.18 | | < LOD | | < LOD | | < LOD | | < LOD | | < LOD |
|  |  | S10 | | < LOD | | < LOD | | < LOD | | 0.022 | | < LOD | | < LOD | | < LOD | | < LOD | | < LOD | | 0.13 | | < LOD | | < LOD | | < LOD | | < LOD | | < LOD |
|  |  | S11 | | < LOD | | < LOD | | < LOD | | 0.016 | | < LOD | | < LOD | | < LOD | | < LOD | | < LOD | | 0.027 | | < LOD | | < LOD | | < LOD | | < LOD | | < LOD |
|  |  | S12 | | < LOD | | < LOD | | < LOD | | 0.020 | | < LOD | | 0.030 | | < LOD | | < LOD | | < LOD | | 0.034 | | < LOD | | < LOD | | < LOD | | < LOD | | < LOD |
|  |  | S13 | | < LOD | | < LOD | | < LOD | | 0.0057 | | < LOD | | 0.041 | | < LOD | | < LOD | | < LOD | | 0.060 | | < LOD | | < LOD | | < LOD | | < LOD | | < LOD |
|  |  | S14 | | < LOD | | < LOD | | < LOD | | 0.0082 | | < LOD | | 0.026 | | < LOD | | 0.023 | | 0.048 | | 0.13 | | 0.053 | | < LOD | | < LOD | | < LOD | | < LOD |
|  |  | S15 | | 0.022 | | < LOD | | < LOD | | 0.061 | | < LOD | | 0.10 | | 0.0080 | | < LOD | | < LOD | | 0.054 | | < LOD | | < LOD | | < LOD | | < LOD | | < LOD |
| December | | S1 | | < LOD | | < LOD | | < LOD | | 0.0087 | | < LOD | | 0.018 | | < LOD | | < LOD | | < LOD | | 0.017 | | < LOD | | < LOD | | < LOD | | < LOD | | < LOD |
|  |  | S2 | | < LOD | | < LOD | | < LOD | | 0.010 | | < LOD | | 0.022 | | 0.0091 | | < LOD | | < LOD | | 0.043 | | < LOD | | < LOD | | < LOD | | < LOD | | < LOD |
|  |  | S3 | | < LOD | | < LOD | | < LOD | | 0.0043 | | < LOD | | 0.019 | | < LOD | | < LO | | < LOD | | 0.014 | | < LOD | | < LOD | | < LOD | | < LOD | | < LOD |
|  |  | S4 | | 0.029 | | 0.045 | | 0.054 | | 0.027 | | < LOD | | 0.093 | | 0.086 | | 0.031 | | < LOD | | 2.21 | | 0.039 | | < LOD | | < LOD | | < LOD | | < LOD |
|  |  | S5 | | < LOD | | < LOD | | < LOD | | 0.0034 | | < LOD | | 0.076 | | < LOD | | 0.036 | | < LOD | | 0.12 | | < LOD | | < LOD | | < LOD | | < LOD | | < LOD |
|  |  | S6 | | < LOD | | < LOD | | < LOD | | 0.0082 | | < LOD | | 0.069 | | < LOD | | 0.037 | | < LOD | | 0.096 | | < LOD | | < LOD | | < LOD | | < LOD | | < LOD |
|  |  | S7 | | < LOD | | < LOD | | < LOD | | 0.0045 | | < LOD | | < LOD | | < LOD | | < LOD | | < LOD | | 0.011 | | < LOD | | < LOD | | < LOD | | < LOD | | < LOD |
|  |  | S8 | | < LOD | | < LOD | | < LOD | | 0.0041 | | < LOD | | 0.012 | | 0.0011 | | < LOD | | < LOD | | 0.012 | | < LOD | | < LOD | | < LOD | | < LOD | | < LOD |
|  |  | S9 | | < LOD | | < LOD | | < LOD | | 0.0065 | | < LOD | | < LOD | | 0.0017 | | < LOD | | < LOD | | 0.023 | | < LOD | | < LOD | | < LOD | | < LOD | | < LOD |
|  |  | S10 | | < LOD | | < LOD | | < LOD | | 0.0041 | | < LOD | | 0.012 | | < LOD | | < LOD | | < LOD | | 0.029 | | < LOD | | < LOD | | < LOD | | < LOD | | < LOD |
|  |  | S11 | | < LOD | | < LOD | | < LOD | | 0.010 | | < LOD | | < LOD | | < LOD | | < LOD | | < LOD | | 0.014 | | < LOD | | < LOD | | < LOD | | < LOD | | < LOD |
|  |  | S12 | | < LOD | | < LOD | | < LOD | | 0.011 | | < LOD | | < LOD | | < LOD | | < LOD | | 0.027 | | 0.079 | | < LOD | | < LOD | | < LOD | | < LOD | | < LOD |
|  |  | S13 | | < LOD | | < LOD | | < LOD | | 0.0037 | | < LOD | | 0.019 | | 0.0032 | | < LOD | | < LOD | | 0.056 | | < LOD | | < LOD | | < LOD | | < LOD | | < LOD |
|  |  | S14 | | < LOD | | < LOD | | < LOD | | 0.0078 | | < LOD | | < LOD | | < LOD | | < LOD | | < LOD | | 0.033 | | < LOD | | < LOD | | < LOD | | < LOD | | < LOD |
|  |  | S15 | | < LOD | | < LOD | | < LOD | | 0.0041 | | < LOD | | < LOD | | < LOD | | < LOD | | < LOD | | 0.016 | | < LOD | | < LOD | | < LOD | | < LOD | | < LOD |

Note, LOD: method limit of detection

**Table S12** Concentrations (ng/g, dw) of PFAS detected in wild fish of the Wuhan section of Yangtze River.

| Samples name | PFBA | PFPeA | PFHxA | PFBS | PFHpA | PFOA | PFHxS | PFNA | PFDA | PFOS | PFUnDA | PFDoA | PFDS | PFTrDA | PFTeDA |
| --- | --- | --- | --- | --- | --- | --- | --- | --- | --- | --- | --- | --- | --- | --- | --- |
| CI1 | < LOD | < LOD | < LOD | < LOD | < LOD | 2.07 | < LOD | 0.42 | 1.28 | 2.47 | 0.28 | 0.20 | < LOD | < LOD | 0.13 |
| CI2 | < LOD | < LOD | < LOD | < LOD | < LOD | 2.97 | < LOD | 0.12 | 0.50 | 6.46 | 1.92 | 0.27 | 0.089 | 0.84 | < LOD |
| CI3 | < LOD | < LOD | 0.081 | < LOD | 0.087 | 0.13 | < LOD | 0.15 | 0.45 | 3.27 | 0.76 | 0.20 | 0.076 | 0.91 | 0.31 |
| CI4 | 0.066 | 0.078 | 0.082 | < LOD | < LOD | < LOD | < LOD | 0.091 | 0.21 | 1.09 | 1.10 | 0.00 | 0.13 | 1.61 | < LOD |
| CI5 | < LOD | < LOD | < LOD | < LOD | 0.11 | 1.89 | < LOD | < LOD | 0.23 | 2.57 | 0.36 | 0.22 | < LOD | 0.94 | 0.35 |
| CI6 | 0.054 | 0.090 | < LOD | < LOD | < LOD | 3.12 | < LOD | 0.076 | 0.48 | 0.98 | 0.34 | 0.44 | 0.13 | 1.23 | 0.43 |
| CI7 | 0.058 | < LOD | < LOD | < LOD | 0.084 | 0.65 | < LOD | < LOD | 0.46 | 1.14 | 0.09 | 0.12 | 0.090 | 0.91 | 0.34 |
| CI8 | < LOD | < LOD | < LOD | < LOD | 0.13 | 0.92 | < LOD | 0.15 | 0.17 | 3.78 | 0.22 | 0.082 | < LOD | 0.12 | 0.25 |
| CI9 | < LOD | < LOD | 0.092 | < LOD | < LOD | 0.75 | < LOD | 0.072 | 0.086 | 4.91 | 2.16 | < LOD | < LOD | 0.12 | < LOD |
| CA1 | < LOD | < LOD | < LOD | < LOD | 0.11 | 5.01 | < LOD | 0.088 | 0.46 | 9.10 | 2.85 | 0.49 | 0.096 | 2.23 | < LOD |
| CA2 | < LOD | < LOD | < LOD | < LOD | 0.11 | 3.23 | < LOD | 0.081 | 1.79 | 3.74 | 1.54 | 0.30 | 1.08 | 1.96 | 0.90 |
| CA3 | 0.077 | < LOD | < LOD | < LOD | 0.11 | 0.78 | < LOD | 0.014 | 0.50 | 11.23 | 2.48 | 0.36 | 0.077 | 0.29 | 0.86 |
| HM1 | 0.049 | 0.080 | 0.086 | < LOD | < LOD | 1.78 | < LOD | 0.14 | 0.80 | 7.46 | 1.70 | 0.40 | 0.44 | 3.71 | 0.68 |
| HM2 | 0.071 | 0.090 | < LOD | < LOD | 0.11 | 1.19 | < LOD | 0.19 | 0.94 | 8.21 | 1.91 | 0.64 | 2.04 | 6.87 | 0.64 |
| HM3 | < LOD | < LOD | < LOD | < LOD | < LOD | 2.00 | < LOD | < LOD | 0.51 | 4.76 | 1.23 | < LOD | 0.13 | 1.70 | < LOD |
| CC1 | < LOD | 0.093 | 0.089 | < LOD | 0.10 | 3.20 | < LOD | < LOD | 0.45 | 9.12 | 2.40 | 0.31 | 0.89 | 3.00 | 0.37 |
| PP1 | < LOD | < LOD | < LOD | < LOD | 0.14 | 1.48 | < LOD | 0.14 | 1.30 | 4.06 | 2.91 | 0.40 | 0.087 | 1.95 | 0.19 |
| CAB1 | 0.098 | < LOD | < LOD | < LOD | < LOD | 3.15 | < LOD | < LOD | 0.61 | 6.08 | 0.92 | 0.12 | 0.075 | < LOD | 0.49 |

Note, LOD: method limit of detection.

**Table S13** Standardized distribution coefficients log Koc (L/kg) for PFAS in the Wuhan section of the Yangtze River.

| Compound | Summer | | | | | Winter | | | | |
| --- | --- | --- | --- | --- | --- | --- | --- | --- | --- | --- |
|  | Min | Max | Mean | Median | | Min | Max | Mean | Median |  |
| PFBA | | -0.24 | 3.96 | 1.68 | 2.21 | | -1.62 | 2.06 | -0.0011 | -0.44 |
| PFPeA | | 0.34 | 2.62 | 1.37 | 1.19 | | 0.049 | 1.50 | 0.73 | 0.72 |
| PFHxA | | 0.50 | 1.59 | 1.03 | 0.90 | | -0.14 | 1.45 | 0.31 | 0.25 |
| PFBS | | -0.30 | 1.74 | 0.73 | 0.78 | | -0.24 | 1.01 | 0.25 | 0.22 |
| PFHpA | | 0.62 | 2.29 | 1.37 | 1.14 | | 0.56 | 1.43 | 1.00 | 1.02 |
| PFOA | | -0.54 | 2.45 | 0.87 | 1.14 | | -0.79 | 1.68 | 0.38 | 0.52 |
| PFHxS | | -0.0066 | 3.02 | 1.94 | 2.19 | | 0.27 | 2.70 | 0.90 | 0.82 |
| PFNA | | 1.14 | 2.13 | 1.71 | 1.75 | | 0.81 | 2.21 | 1.37 | 1.36 |
| PFDA | | 2.21 | 3.20 | 2.59 | 2.49 | | 1.83 | 2.63 | 2.23 | 2.28 |
| PFOS | | 0.88 | 3.93 | 2.31 | 2.28 | | 0.74 | 4.10 | 1.84 | 1.74 |
| PFUnDA | | 2.21 | 3.24 | 2.60 | 2.49 | | 1.79 | 3.28 | 2.24 | 2.21 |
| PFDoA | | 2.12 | 3.02 | 2.46 | 2.37 | | 1.79 | 2.63 | 2.18 | 2.21 |
| PFDS | | 2.21 | 3.02 | 2.43 | 2.29 | | 1.79 | 2.63 | 2.18 | 2.21 |
| PFTrDA | | 2.12 | 3.02 | 2.46 | 2.37 | | 1.79 | 2.63 | 2.18 | 2.21 |
| PFTeDA | | 2.12 | 3.02 | 2.46 | 2.37 | | 1.79 | 2.63 | 2.18 | 2.21 |

**Table S14** Exposure risk assessment of PFAS in wild fish in the Wuhan section of Yangtze River.

| Compound | PFBA | PFPeA | PFHxA | PFBS | PFHpA | PFOA | PFHxS | PFNA | PFDA | PFOS | PFUnDA | PFDoA | PFDS | PFTrDA | PFTeDA |
| --- | --- | --- | --- | --- | --- | --- | --- | --- | --- | --- | --- | --- | --- | --- | --- |
| HR | | | | | | | | | | | | | | | |
| CI | 2.78×10^-6^ | 2.78×10^-5^ | 2.78×10^-5^ | 1.98×10^-6^ | 6.03×10^-4^ | 0.014 | 7.30×10^-4^ | 1.40×10^-3^ | 0.0056 | 0.021 | 0.0056 | 0.0030 | 1.16×10^-3^ | 0.014 | 0.0039 |
| CA | 2.78×10^-6^ | 2.78×10^-5^ | 2.78×10^-5^ | 1.98×10^-6^ | 9.01×10^-4^ | 0.050 | 7.30×10^-4^ | 1.25×10^-3^ | 0.0062 | 0.073 | 0.038 | 0.0055 | 1.48×10^-3^ | 0.030 | 0.0133 |
| HM | 9.07×10^-6^ | 2.96×10^-5^ | 2.78×10^-5^ | 1.98×10^-6^ | 6.03×10^-4^ | 0.027 | 7.30×10^-4^ | 0.0021 | 0.0099 | 0.060 | 0.026 | 0.0062 | 0.0067 | 0.057 | 0.0098 |
| CC | 2.78×10^-6^ | 3.44×10^-5^ | 3.29×10^-5^ | 1.98×10^-6^ | 8.29×10^-4^ | 0.049 | 7.30×10^-4^ | 1.16×10^-3^ | 0.0056 | 0.073 | 0.037 | 0.0047 | 0.014 | 0.046 | 0.0056 |
| PP | 2.78×10^-6^ | 2.78×10^-5^ | 2.78×10^-5^ | 1.98×10^-6^ | 1.09×10^-4^ | 0.023 | 7.30×10^-4^ | 0.0021 | 0.016 | 0.033 | 0.045 | 0.0062 | 1.34×10^-3^ | 0.030 | 0.0029 |
| CAB | 1.81×10^-5^ | 2.78×10^-5^ | 2.78×10^-5^ | 1.98×10^-6^ | 6.03×10^-4^ | 0.049 | 7.30×10^-4^ | 1.16×10^-3^ | 0.0076 | 0.049 | 0.014 | 0.0019 | 1.16×10^-3^ | 1.16×10^-3^ | 0.0076 |

**Table S15** Concentrations (ng/L) and detection frequencies of PFAS in water of the Wuhan section of the Yangtze River in summer (n=15) and winter (n=15).

| Compound | Summer | | | | | | Winter | | | |
| --- | --- | --- | --- | --- | --- | --- | --- | --- | --- | --- |
|  | Min | Max | Mean | DF（%） | Min | | Max | Mean | DF（%） |  |
| PFBA | | <LOD | 1.62 | 0.54 | 40.0 | <LOD | | 12.56 | 3.26 | 80.0 |
| PFPeA | | <LOD | 1.89 | 0.75 | 73.3 | 0.45 | | 1.74 | 0.90 | 100.0 |
| PFHxA | | 0.59 | 1.29 | 0.79 | 100.0 | 1.84 | | 2.63 | 2.22 | 100.0 |
| PFBS | | 0.92 | 35.50 | 4.45 | 100.0 | 1.87 | | 5.37 | 3.03 | 100.0 |
| PFHpA | | 0.12 | 0.97 | 0.42 | 100.0 | 0.30 | | 0.55 | 0.39 | 100.0 |
| PFOA | | 1.51 | 2.82 | 2.03 | 100.0 | 1.89 | | 3.79 | 2.38 | 100.0 |
| PFHxS | | <LOD | 0.83 | 0.096 | 80.0 | 0.10 | | 0.48 | 0.18 | 100.0 |
| PFNA | | 0.10 | 0.30 | 0.20 | 100.0 | 0.18 | | 0.32 | 0.24 | 100.0 |
| PFDA | | <LOD | <LOD | <LOD | - | <LOD | | <LOD | <LOD | - |
| PFOS | | 0.16 | 1.51 | 0.44 | 100.0 | 0.21 | | 0.97 | 0.47 | 100.0 |
| PFUnDA | | <LOD | <LOD | <LOD | - | <LOD | | <LOD | <LOD | - |
| PFDoA | | <LOD | <LOD | <LOD | - | <LOD | | <LOD | <LOD | - |
| PFDS | | <LOD | 0.13 | 0.032 | 6.7 | <LOD | | <LOD | <LOD | - |
| PFTrDA | | <LOD | <LOD | <LOD | - | <LOD | | <LOD | <LOD | - |
| PFTeDA | | <LOD | <LOD | <LOD | - | <LOD | | <LOD | <LOD | - |
| Total | | 5.26 | 43.39 | 9.88 | 53.3 | 8.44 | | 22.96 | 13.23 | 58.7 |

**Table S16** Concentrations (ng/g, dw) and detection frequencies of PFAS in sediments of the Wuhan section of the Yangtze River in summer (n=15) and winter (n=15).

| Compound | Summer | | | | | Winter | | | |
| --- | --- | --- | --- | --- | --- | --- | --- | --- | --- |
|  | Min | Max | Mean | DF（%） | Min | Max | Mean | DF（%） |  |
| PFBA | | < LOD | 0.026 | 0.0041 | 13.3 | < LOD | 0.029 | 0.0029 | 6.7 |
| PFPeA | | < LOD | < LOD | < LOD | - | < LOD | 0.045 | 0.0077 | 6.7 |
| PFHxA | | < LOD | 0.035 | 0.0070 | 6.7 | < LOD | 0.054 | 0.0082 | 6.7 |
| PFBS | | < LOD | 0.061 | 0.013 | 93.3 | 0.0034 | 0.027 | 0.0079 | 100.0 |
| PFHpA | | < LOD | 0.050 | 0.0080 | 6.7 | < LOD | < LOD | < LOD | - |
| PFOA | | < LOD | 0.66 | 0.066 | 60.0 | < LOD | 0.093 | 0.023 | 60.0 |
| PFHxS | | < LOD | 0.041 | 0.0058 | 33.3 | < LOD | 0.086 | 0.0074 | 33.3 |
| PFNA | | < LOD | 0.026 | 0.0087 | 20.0 | < LOD | 0.037 | 0.011 | 20.0 |
| PFDA | | < LOD | 0.048 | 0.010 | 13.3 | < LOD | 0.027 | 0.0065 | 6.7 |
| PFOS | | < LOD | 3.18 | 0.28 | 93.3 | 0.010 | 2.21 | 0.18 | 100.0 |
| PFUnDA | | < LOD | 0.053 | 0.011 | 13.3 | < LOD | 0.039 | 0.0073 | 6.7 |
| PFDoA | | < LOD | < LOD | < LOD | - | < LOD | < LOD | < LOD | - |
| PFDS | | < LOD | 0.0073 | 0.0052 | 6.7 | < LOD | < LOD | < LOD | - |
| PFTrDA | | < LOD | < LOD | < LOD | - | < LOD | < LOD | < LOD | - |
| PFTeDA | | < LOD | < LOD | < LOD | - | < LOD | < LOD | < LOD | - |
| Total | | 0.055 | 4.02 | 0.44 | 24.0 | 0.068 | 2.64 | 0.29 | 23.1 |

**Table S17** The average daily fluxes (kg/d) in different sites and seasons.

| **Site** | **Time** | **PFBA** | **PFPeA** | **PFHxA** | **PFBS** | **PFHpA** | **PFOA** | **PFHxS** | **PFNA** | **PFOS** | **PFDS** | **TOTAL** |
| --- | --- | --- | --- | --- | --- | --- | --- | --- | --- | --- | --- | --- |
| S1 | 2022.6-8 | 2.49 | 0.97 | 1.53 | 2.36 | 0.542 | 5.55 | 0.029 | 0.47 | 0.78 | 0.21 | 14.93 |
|  | 2022.9-11 | 0.49 | 0.81 | 1.33 | 1.76 | 0.292 | 1.45 | 0.051 | 0.18 | 0.26 | 0.038 | 6.67 |
|  | 2022.12-2023.2 | 1.03 | 1.07 | 2.12 | 1.97 | 0.27 | 2.13 | 0.066 | 0.12 | 0.26 | 0.019 | 9.06 |
|  | 2023.3-5 | 0.0049 | 0.87 | 3.10 | 5.17 | 0.44 | 2.36 | 0.040 | 0.26 | 0.25 | 0.040 | 12.54 |
| S8 | 2022.6-8 | 3.27 | 2.96 | 1.69 | 2.56 | 2.33 | 4.50 | 0.16 | 0.58 | 1.49 | 0.077 | 19.62 |
|  | 2022.9-11 | 2.18 | 0.89 | 1.26 | 1.25 | 0.34 | 1.71 | 0.066 | 0.25 | 0.46 | 0.034 | 8.44 |
|  | 2022.12-2023.2 | 2.30 | 0.96 | 1.92 | 2.10 | 0.34 | 2.19 | 0.18 | 0.17 | 0.29 | 0.020 | 10.47 |
|  | 2023.3-5 | 0.0052 | 2.42 | 2.77 | 13.09 | 0.58 | 2.69 | 0.41 | 0.50 | 0.61 | 0.026 | 23.10 |
| S15 | 2022.6-8 | 1.81 | 0.48 | 1.87 | 5.73 | 1.97 | 5.92 | 0.22 | 0.75 | 0.88 | 0.078 | 19.71 |
|  | 2022.9-11 | 1.70 | 1.70 | 1.75 | 1.58 | 0.29 | 1.86 | 0.11 | 0.20 | 0.31 | 0.023 | 9.52 |
|  | 2022.12-2023.2 | 1.78 | 0.89 | 2.27 | 3.49 | 0.31 | 2.12 | 0.17 | 0.11 | 0.51 | 0.020 | 11.67 |
|  | 2023.3-5 | 0.63 | 1.14 | 4.40 | 11.40 | 0.48 | 3.13 | 0.22 | 0.23 | 0.40 | 0.030 | 22.04 |

**Table S18** The annual fluxes (t/a) in different sites.

| **Site** | **PFBA** | **PFPeA** | **PFHxA** | **PFBS** | **PFHpA** | **PFOA** | **PFHxS** | **PFNA** | **PFOS** | **PFDS** | **TOTAL** |
| --- | --- | --- | --- | --- | --- | --- | --- | --- | --- | --- | --- |
| S1 | 0.35 | 0.50 | 1.00 | 1.69 | 0.18 | 1.18 | 0.027 | 0.11 | 0.15 | 0.024 | 5.22 |
| S8 | 0.85 | 0.78 | 0.91 | 2.65 | 0.28 | 1.15 | 0.11 | 0.15 | 0.26 | 0.015 | 7.15 |
| S15 | 0.72 | 0.60 | 1.30 | 3.23 | 0.25 | 1.39 | 0.098 | 0.12 | 0.22 | 0.013 | 7.95 |

**Table S19** Bioaccumulation Factor (log BAF) of six common fish species in the Wuhan section of Yangtze River.

| Fish species | CI | CA | HM | CC | PP | CAB |
| --- | --- | --- | --- | --- | --- | --- |
| PFBA | 1.18 | 1.47 | 1.38 | 1.07 | 1.07 | 1.86 |
| PFPeA | 1.90 | 2.12 | 1.67 | 1.90 | 1.81 | 1.60 |
| PFHxA | 2.03 | 1.99 | 1.92 | 2.03 | 1.96 | 2.04 |
| PFBS | 1.16 | 1.68 | 1.03 | 1.21 | 1.21 | 1.17 |
| PFHpA | 2.30 | 2.89 | 2.08 | 2.54 | 2.66 | 1.92 |
| PFOA | 2.62 | 3.48 | 2.78 | 3.22 | 2.89 | 3.32 |
| PFHxS | 3.12 | 2.71 | 2.32 | 2.68 | 2.68 | 2.54 |
| PFNA | 2.86 | 2.91 | 2.74 | 2.71 | 2.97 | 2.54 |
| PFDA | 4.12 | 4.48 | 4.46 | 4.26 | 4.72 | 4.39 |
| PFOS | 4.00 | 4.53 | 3.83 | 4.48 | 4.13 | 4.12 |
| PFUnDA | 4.31 | 4.95 | 4.80 | 4.98 | 5.07 | 4.57 |
| PFDoA | 3.80 | 4.18 | 4.03 | 4.09 | 4.20 | 3.69 |
| PFDS | 3.55 | 3.90 | 4.29 | 4.55 | 3.54 | 3.48 |
| PFTrDA | 4.28 | 4.63 | 5.15 | 5.08 | 4.89 | 3.48 |
| PFTeDA | 3.86 | 4.19 | 4.11 | 4.16 | 3.88 | 4.29 |

CI: *Ctenopharyngodon idellus*, CA: *Carassius auratus*, HM: *Hypophthalmichthys molitrix*, CC: *Cyprinus carpio*, PP: *Parabramis pekinensis*, CAB: *Culter alburnus Basilewsky*.


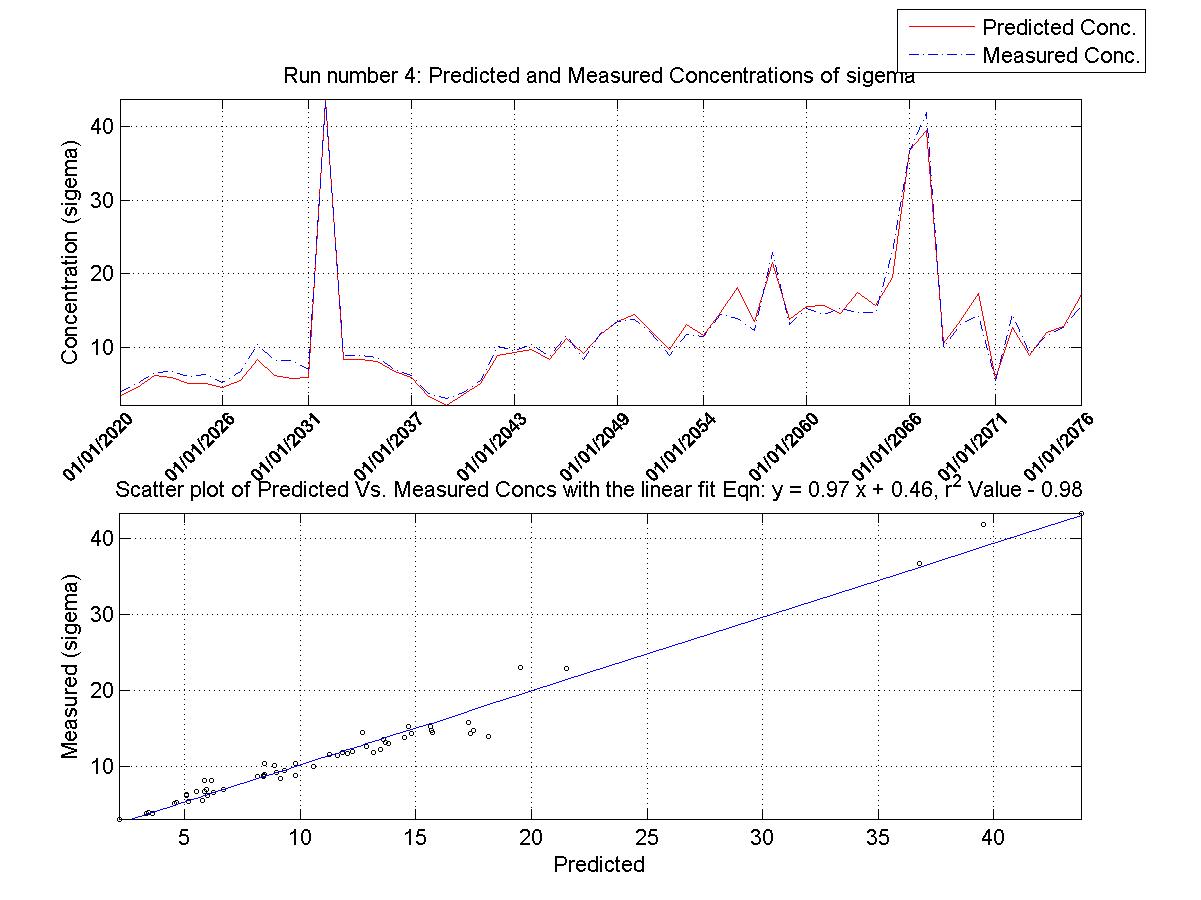

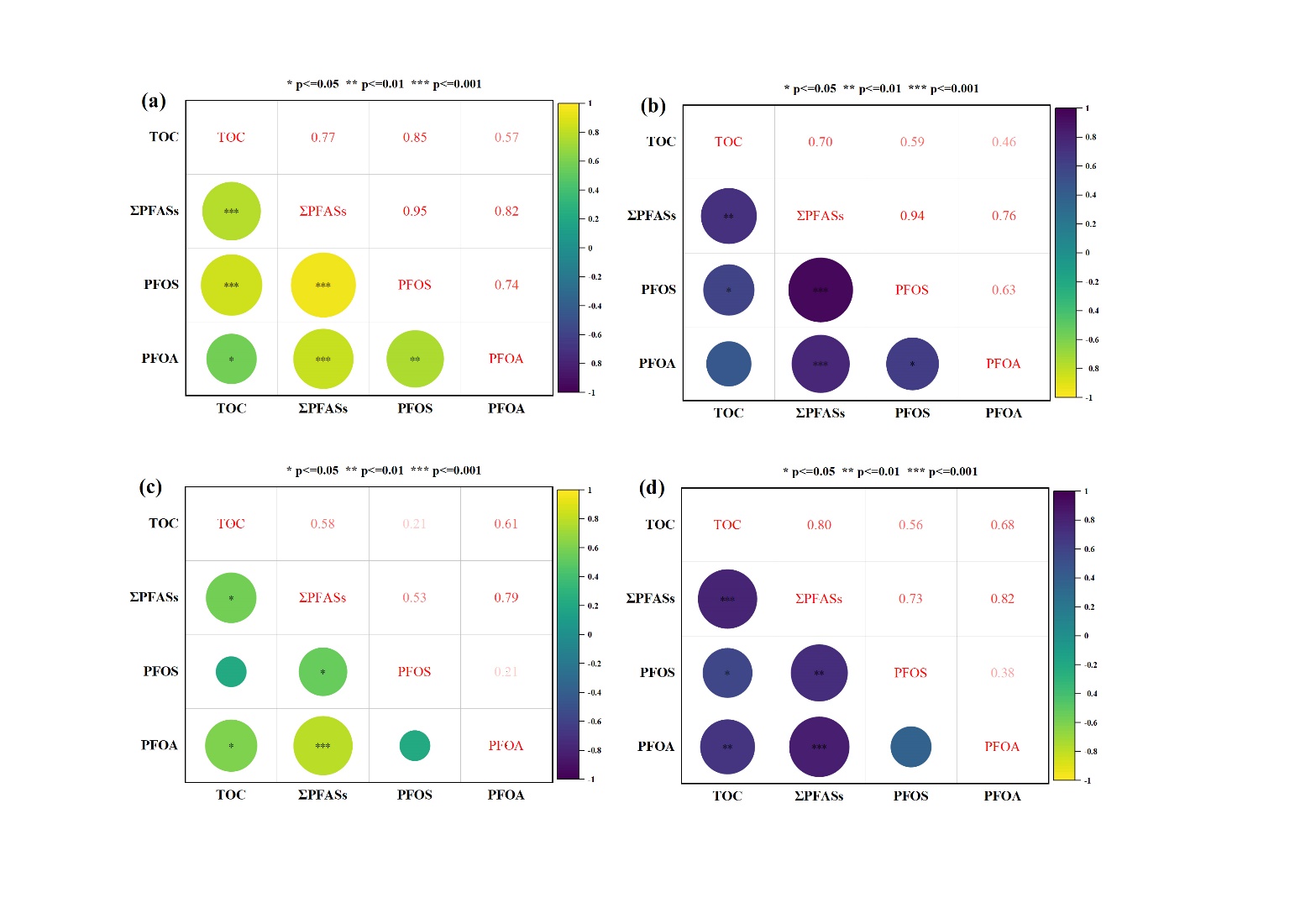
**Fig. S1.** The fitting curve predicted by Unmix model.

**Fig. S2.** Spearman’s correlation of PFAS and TOC in sediment in summer (a), and in winter (b).

**
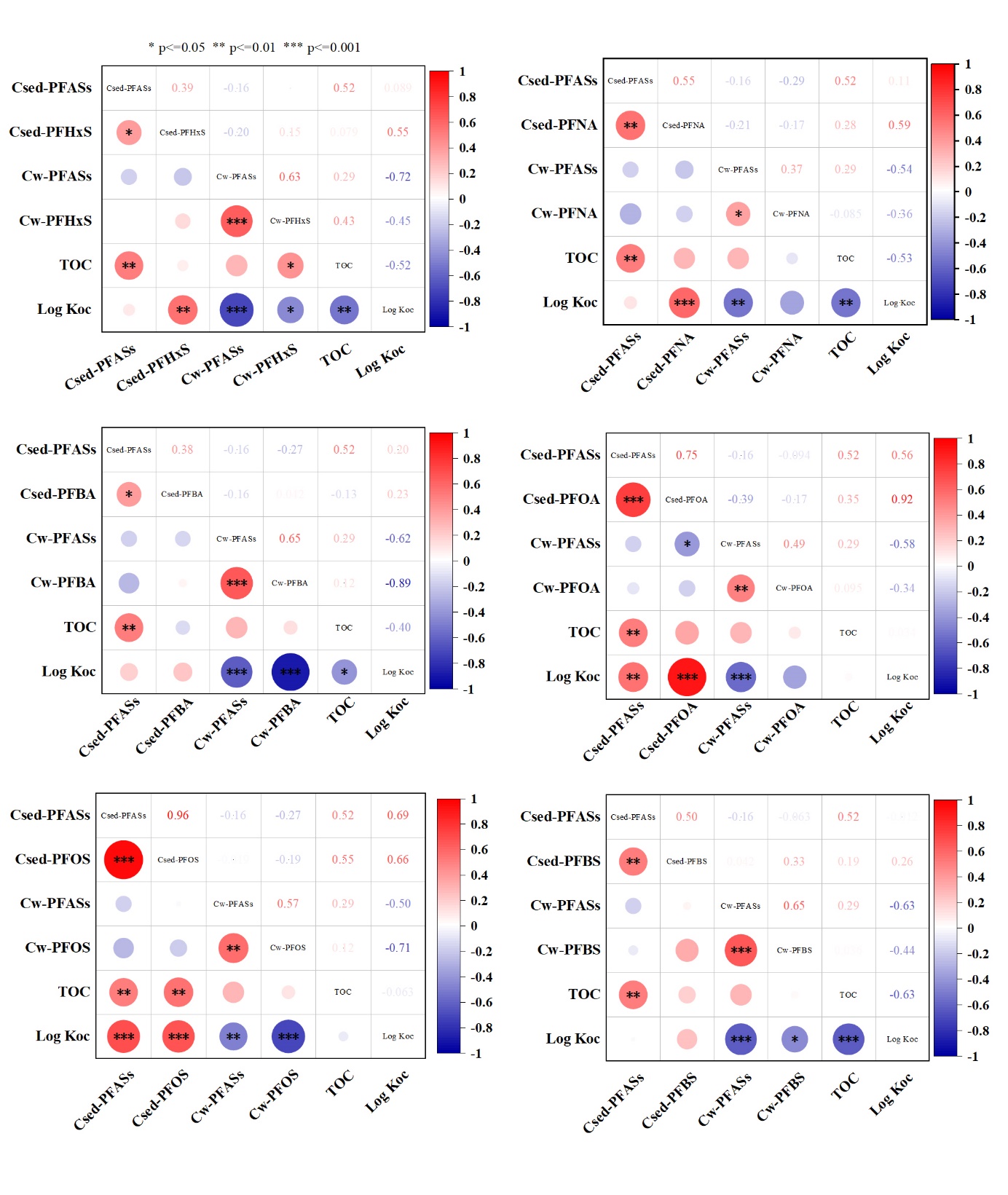
Fig. S3.** The correlation between the log K_OC_ of several substance and concentrations in sediment and water, TOC in sediment.

**
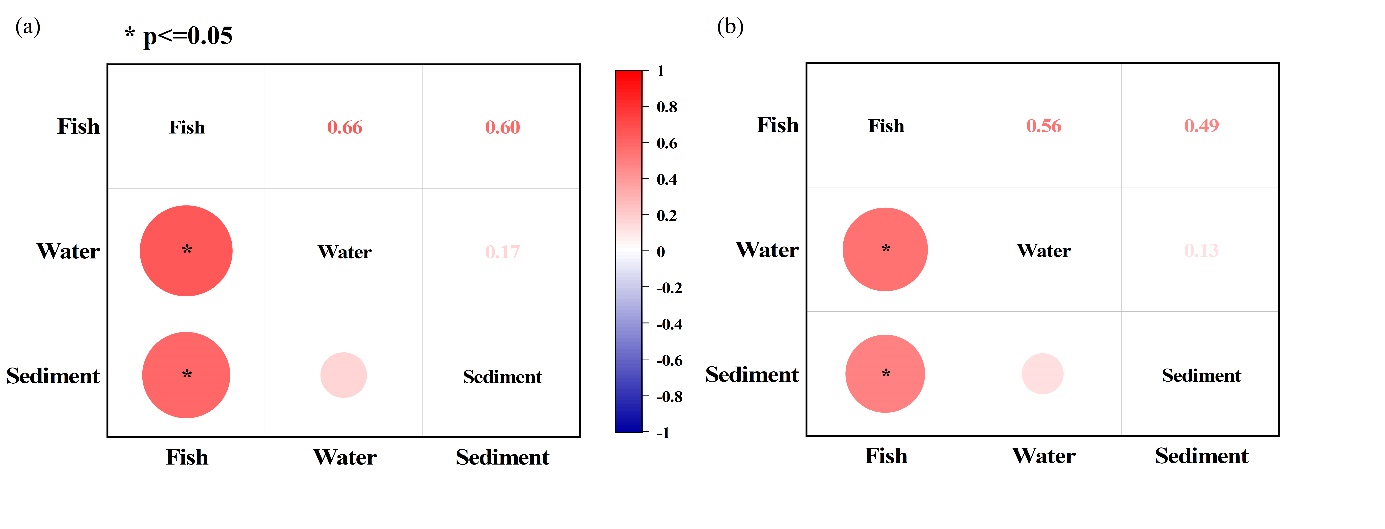
Fig. S4.** The correlation of the total concentration of 15 PFAS (a) and PFOS (b) in different media.

**Reference**

[1] Wallis D J. Source apportionment of serum PFASs in two highly exposed communities[J]. Science of the Total Environment, 2023.

[2] Zhang X, Lohmann R, Sunderland E M. Poly- and Perfluoroalkyl Substances in Seawater and Plankton from the Northwestern Atlantic Margin[J]. Environmental Science & Technology, 2019, 53(21): 12348-12356.

[3] Ng K, Alygizakis N, Androulakakis A, et al. Target and suspect screening of 4777 per- and polyfluoroalkyl substances (PFAS) in river water, wastewater, groundwater and biota samples in the Danube River Basin[J]. Journal of Hazardous Materials, 2022, 436: 129276.
